# Chemical Control of the Dimensionality of the Octahedral Network of Solar Absorbers from the CuI–AgI–BiI_3_ Phase Space by Synthesis of 3D CuAgBiI_5_

**DOI:** 10.1021/acs.inorgchem.1c02773

**Published:** 2021-11-09

**Authors:** Harry C. Sansom, Leonardo R. V. Buizza, Marco Zanella, James T. Gibbon, Michael J. Pitcher, Matthew S. Dyer, Troy D. Manning, Vinod R. Dhanak, Laura M. Herz, Henry J. Snaith, John B. Claridge, Matthew J. Rosseinsky

**Affiliations:** † Department of Chemistry, Materials Innovation Factory, University of Liverpool, 51 Oxford Street, Liverpool L7 3NY, U.K.; ‡ Clarendon Laboratory, Department of Physics, University of Oxford, Parks Road, Oxford OX1 3PU, U.K.; § Stephenson Institute for Renewable Energy and Department of Physics, University of Liverpool, Oxford Street, Liverpool L69 7ZF, U.K.

## Abstract

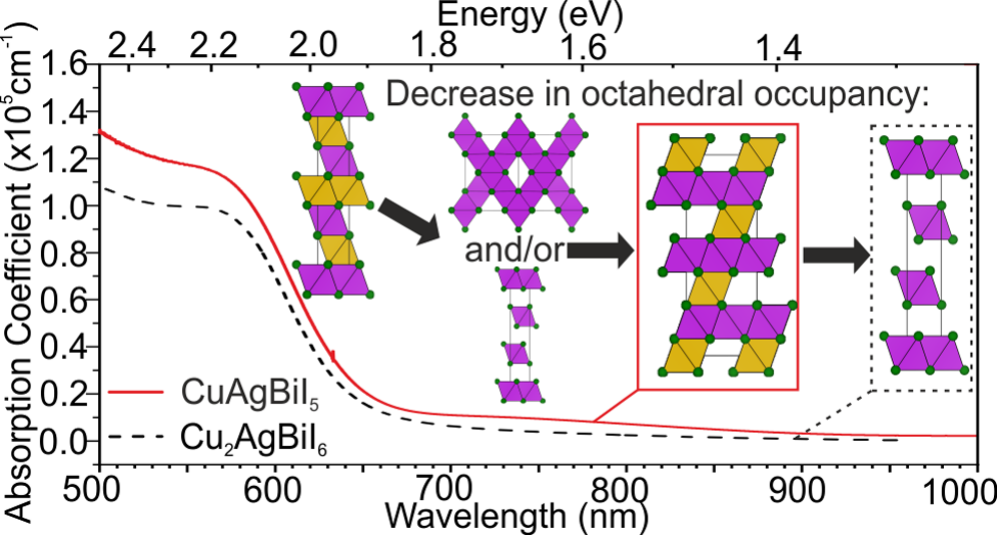

A newly reported compound, CuAgBiI_5_, is synthesized as powder, crystals, and thin films. The structure consists of a 3D octahedral Ag^+^/Bi^3+^ network as in spinel, but occupancy of the tetrahedral interstitials by Cu^+^ differs from those in spinel. The 3D octahedral network of CuAgBiI_5_ allows us to identify a relationship between octahedral site occupancy (composition) and octahedral motif (structure) across the whole CuI–AgI–BiI_3_ phase field, giving the ability to chemically control structural dimensionality. To investigate composition–structure–property relationships, we compare the basic optoelectronic properties of CuAgBiI_5_ with those of Cu_2_AgBiI_6_ (which has a 2D octahedral network) and reveal a surprisingly low sensitivity to the dimensionality of the octahedral network. The absorption onset of CuAgBiI_5_ (2.02 eV) barely changes compared with that of Cu_2_AgBiI_6_ (2.06 eV) indicating no obvious signs of an increase in charge confinement. Such behavior contrasts with that for lead halide perovskites which show clear confinement effects upon lowering dimensionality of the octahedral network from 3D to 2D. Changes in photoluminescence spectra and lifetimes between the two compounds mostly derive from the difference in extrinsic defect densities rather than intrinsic effects. While both materials show good stability, bulk CuAgBiI_5_ powder samples are found to be more sensitive to degradation under solar irradiation compared to Cu_2_AgBiI_6_.

## Abstract 2

We describe a way to chemically control the octahedral network of potentially useful photovoltaic solar absorbers in the CuI−AgI−BiI_3_ phase space by the synthesis of CuAgBiI_5_ with a 3D octahedral network. We compare the photostability of CuAgBiI_5_ bulk samples and the absorption coefficient and photoluminescence of solution processed thin films with those of Cu_2_AgBiI_6_, which has a 2D octahedral network. This helps to understand structure−property relationships to direct further materials optimization.

## Introduction

Ternary and quaternary compounds from the CuI–AgI–BiI_3_ phase space show huge potential for photovoltaics due to their suitable band gaps (1.67–2.06 eV) with very high absorption coefficients exceeding 10^5^ cm^–1^ and low excitonic binding energies (∼25 meV) that arise from their stable Bi^3+^ iodide octahedral network in a close packed iodide sublattice.^1−4^ In contrast, the double perovskite Cs_2_AgBiBr_6_ does not strongly absorb light at energies below its excitonic absorption peak at 2.8 eV and direct band gap at 3.03 eV,^5,6^ and the hypothetical compound Cs_2_AgBiI_6_, which would likely have a narrower band gap, is not a stable phase.^7^ Therefore, the materials from the CuI–AgI–BiI_3_ phase space fill an important gap in “lead-free” metal-halide materials capability. They also overcome the compromises of the wide band gap hybrid lead halide perovskites *A*Pb(Br_*x*_I_1–*x*_)_3_. Although the hybrid lead halide perovskites can be used to make highly efficient photovoltaic devices, they can suffer from halide segregation under illumination (due to competing phases arising from mixing of the halides)^8,9^ and low thermal stability (due to the presence of organic cations)^10,11^ and have to be carefully managed due to known toxicological issues with lead. In comparison, the CuI–AgI–BiI_3_ materials contain a single halide, are entirely inorganic, and are lead-free. Photovoltaic devices utilizing CuI–AgI–BiI_3_ materials as the solar absorbers have reached over 5% power conversion efficiencies (PCEs).^12^ Further improvements for devices using existing materials are expected to come from optimizing device architecture and transport layers and passivation techniques. However, further materials development is also crucial for the realization of efficient devices, and to achieve this, there is the need to understand the composition–structure–property relationships across the CuI–AgI–BiI_3_ phase space. The CuI–AgI–BiI_3_ compounds do not form perovskites. We have previously reported the structural perspective showing that the CuI–AgI–BiI_3_ compounds have an uninterrupted close-packed anion sublattice, whereas perovskites have a close-packed anion sublattice which is interrupted by the large *A*-site cations.^1^ Here, we report the quaternary CuAgBiI_5_ which shows how the widely variable composition of materials from the CuI–AgI–BiI_3_ phase space can be used to select the dimensionality of the octahedral motif. This is an important relationship to understand because the properties of the lead halide perovskites drastically change depending on whether the octahedral network is 2D or 3D. The 2D lead halide perovskites are more stable, but the lower dimensionality of the octahedral networks results in both wider band gaps and highly confined charge carriers leading to excitons, which are less desirable for photovoltaic applications.^13,14^ We compare the properties of the three-dimensional (3D) octahedral network of CuAgBiI_5_ with the two-dimensional (2D) octahedral network of previously reported Cu_2_AgBiI_6_.

## CuI–AgI–BiI_3_ Phase Space

**Figure 1:**
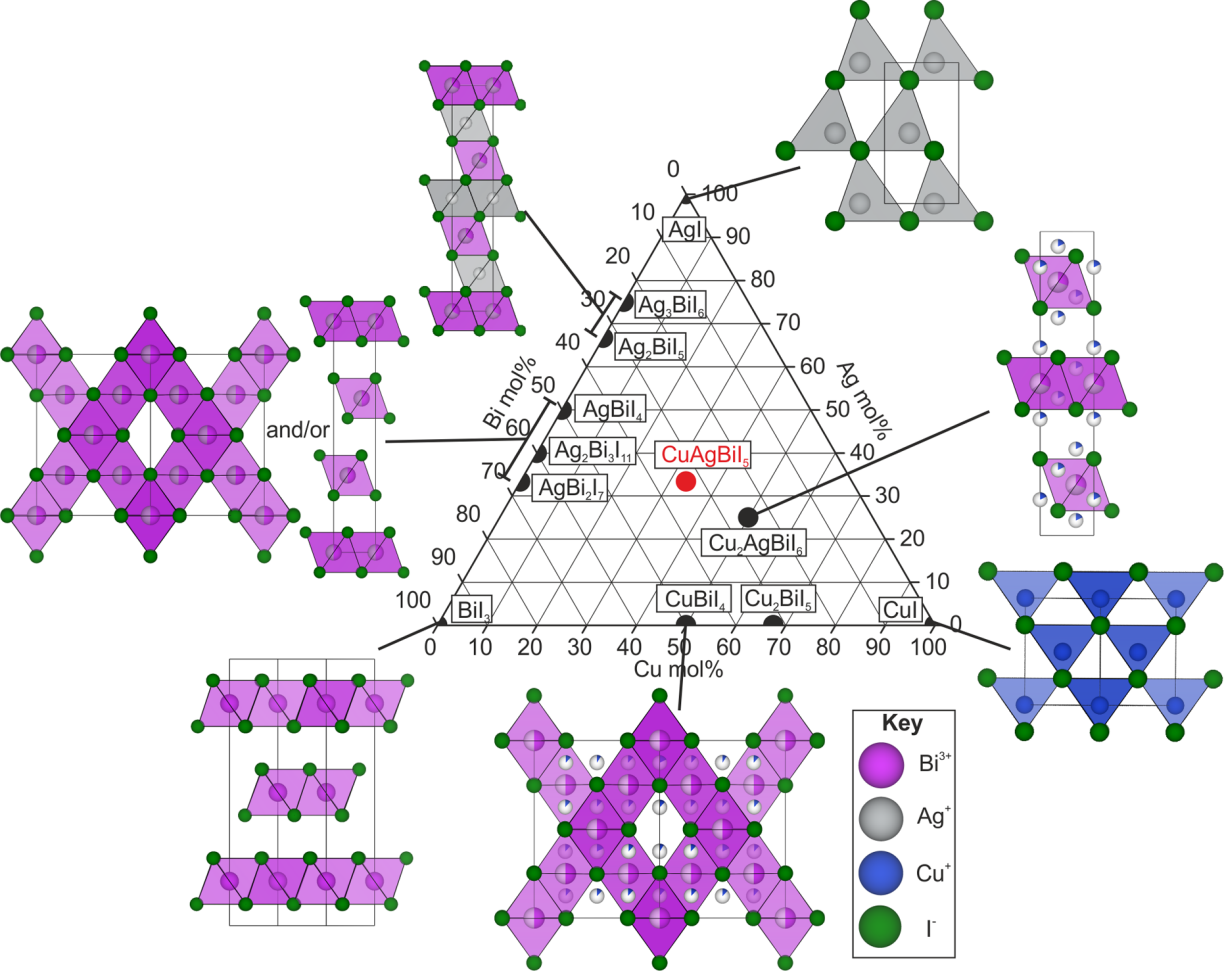
Reported structures in the CuI–AgI–BiI_3_ phase space: binaries CuI, AgI, and BiI_3_;^15−17^ ternaries Ag_3_BiI_6_, Ag_2_BiI_5_, AgBiI_4_, AgBi_2_I_7_, Ag_2_Bi_3_I_11_, CuBiI_4_, and Cu_2_BiI_5_;^1,18−22^ and quaternary Cu_2_AgBiI_6_.^2^ We have also added the compound we report here, CuAgBiI_5_. As discussed in the text, all these compounds consist of a close-packed iodide sublattice with varying arrangements of the cations filling the octahedral and tetrahedral interstitial sites to form the structures shown.

The CuI–AgI–BiI_3_ phase space is shown in Figure 1. It is mapped out by known binary compounds CuI, AgI, and BiI_3_ at the corners,^15−17^ ternary Ag–Bi–I (Ag_3_BiI_6_, Ag_2_BiI_5_, AgBiI_4_, AgBi_2_I_7_, Ag_2_Bi_3_I_11_)^1,18−21^ and Cu–Bi–I (CuBiI_4_, Cu_2_BiI_5_)^22^ compounds on the edges, and a quaternary Cu–Ag–Bi–I (Cu_2_AgBiI_6_)^2^ compound in the enclosed area. We have also added the new compound described in this study, CuAgBiI_5_. The Ag–Bi–I materials have been the most studied for photovoltaics. Low temperature synthesis and processing techniques lead to the formation of Ag_2_BiI_5_ or AgBi_2_I_7_, whereas phases Ag_3_BiI_6_, AgBiI_4_, and Ag_2_Bi_3_I_11_ are attainable via high temperature routes.^4^ The Ag-rich compounds Ag_2_BiI_5_ and Ag_3_BiI_6_, the latter of which is a mixture of Ag_2_BiI_5_ and AgI when solution processed into films, are reported to perform better in devices.^4^ The record device PCE of 5.56% uses a solar absorber with nominal composition Ag_3_BiI_5.92_S_0.04_—a small sulfide substitution in Ag_3_BiI_6_.^12^ CuBiI_4_ has been processed into devices with a maximum PCE of 1.1%;^23,24^ however, we previously found this composition to be an unstable phase, and it decomposes back into CuI and BiI_3_ at room temperature.^2^ To stabilize a Cu-containing material, we previously synthesized Cu_2_AgBiI_6_ and fabricated a preliminary device with a PCE of 0.43%.^2,25^ The band gap was found to be 2.06(1) eV, which was modeled to pair efficiently with a crystalline silicon solar absorber in a lead-free tandem cell. Little is known about the other Cu-containing compound Cu_2_BiI_5_, which has been reported to crystallize in a hexagonal unit cell,^22^ with no crystal structural solution or devices reported yet; but it has a band gap of 1.53–1.74 eV.^26^

## Common Structural Features

**Figure 2:**
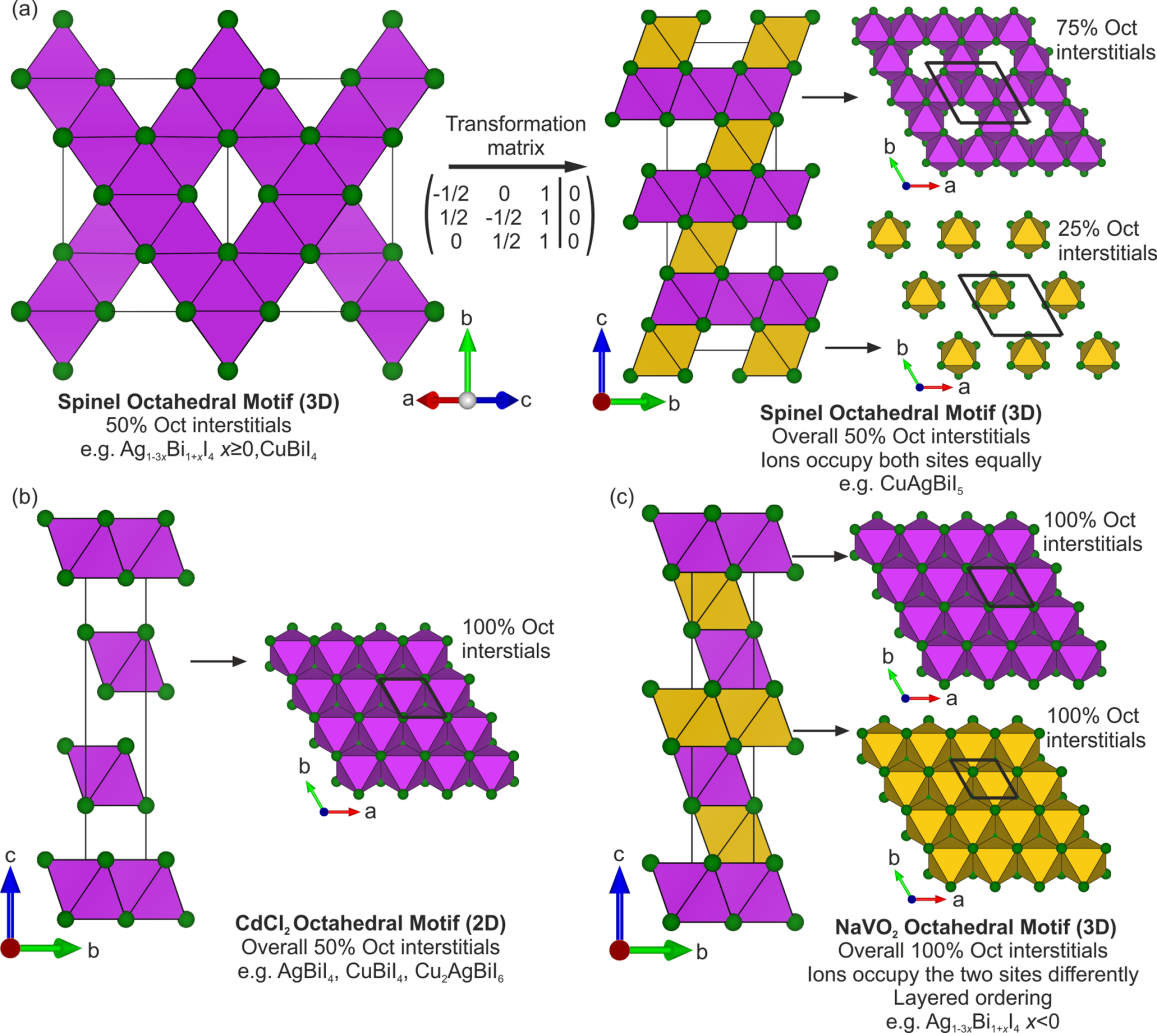
Octahedral (Oct) motifs of ternary and quaternary Cu–Ag–Bi–I compounds.^1,2,18−22^ (a) The spinel Oct motif of *x* ≥ 0 Ag_1–3*x*_Bi_1+*x*_I_4_ and CuBiI_4_ consists of a 3D edge-sharing Oct motif present in spinel (Figure S12) with half of the Oct interstitials occupied. Using the transformation matrix shown, it can be represented in a trigonal unit cell where it can be considered as alternating between layers of 75% and 25% Oct interstitials occupied, maintaining the overall half Oct site occupancy. The reduction in symmetry splits the octahedra into two different sites (depicted by the purple and yellow colors). This representation is necessary to refine the rhombohedral strain of CuAgBiI_5_. (b) The 2D CdCl_2_ Oct motif consists of alternating between layers of full Oct interstitial occupancy and vacant layers, giving overall half Oct interstitial occupancy. (c) The NaVO_2_ Oct motif consists of every possible Oct interstitial being occupied, with layered ordering. The layered ordering means that the layers alternate between two different Oct sites. Unit cells are drawn with solid black lines.

**Figure 3:**
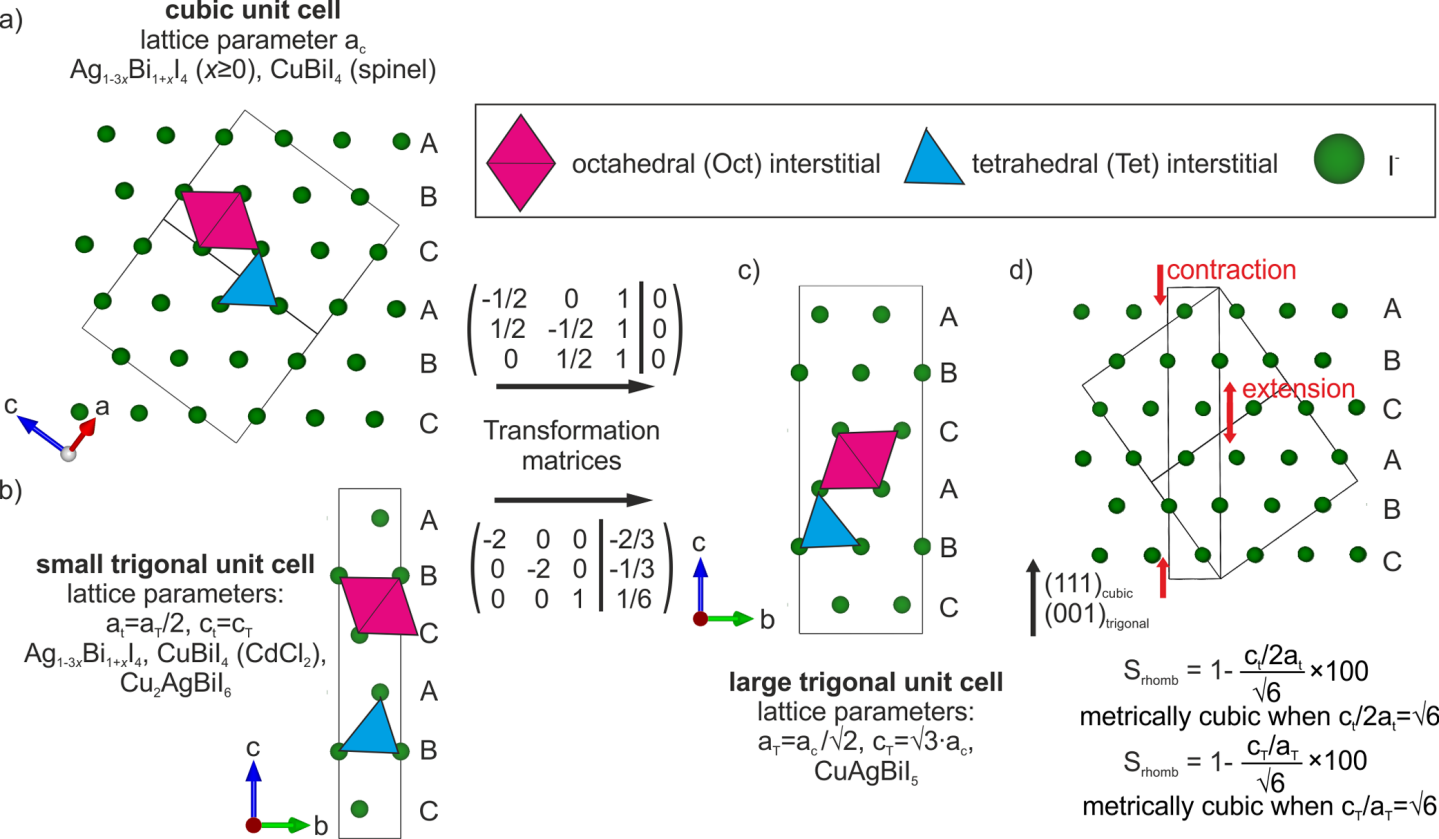
Unit cell relationships, close-packed anion sublattices, and examples of octahedral (Oct) and tetrahedal (Tet) interstitials for the reported ternary and quaternary Cu–Ag–Bi–I compounds.^1,2,18−22^ (a) The cubic unit cell of Ag_1–3*x*_Bi_1+*x*_I_4_ (*x* ≥ 0) and CuBiI_4_ (spinel) and (b) the small trigonal unit cell of Ag_1–3*x*_Bi_1+*x*_I_4_, CuBiI_4_ (CdCl_2_), and Cu_2_AgBiI_6_.^1,18−22^ It is helpful to directly compare the structures by transforming both these cells into a large trigonal unit cell using the transformation matrices shown. (c) This large trigonal cell has *a* and *b* directions double that of the small trigonal cell with a volume four times as large and is √3/2 the volume of the cubic unit cell. The quaternary compound CuAgBiI_5_ crystallizes in the large trigonal unit cell. (d) Rhombohedral strain defined for the cases of the small and large trigonal cells. It can be defined as the extension or contraction of the otherwise cubic structure along the body diagonal (111)_cubic_, shown by the red arrows.

**Figure 4:**
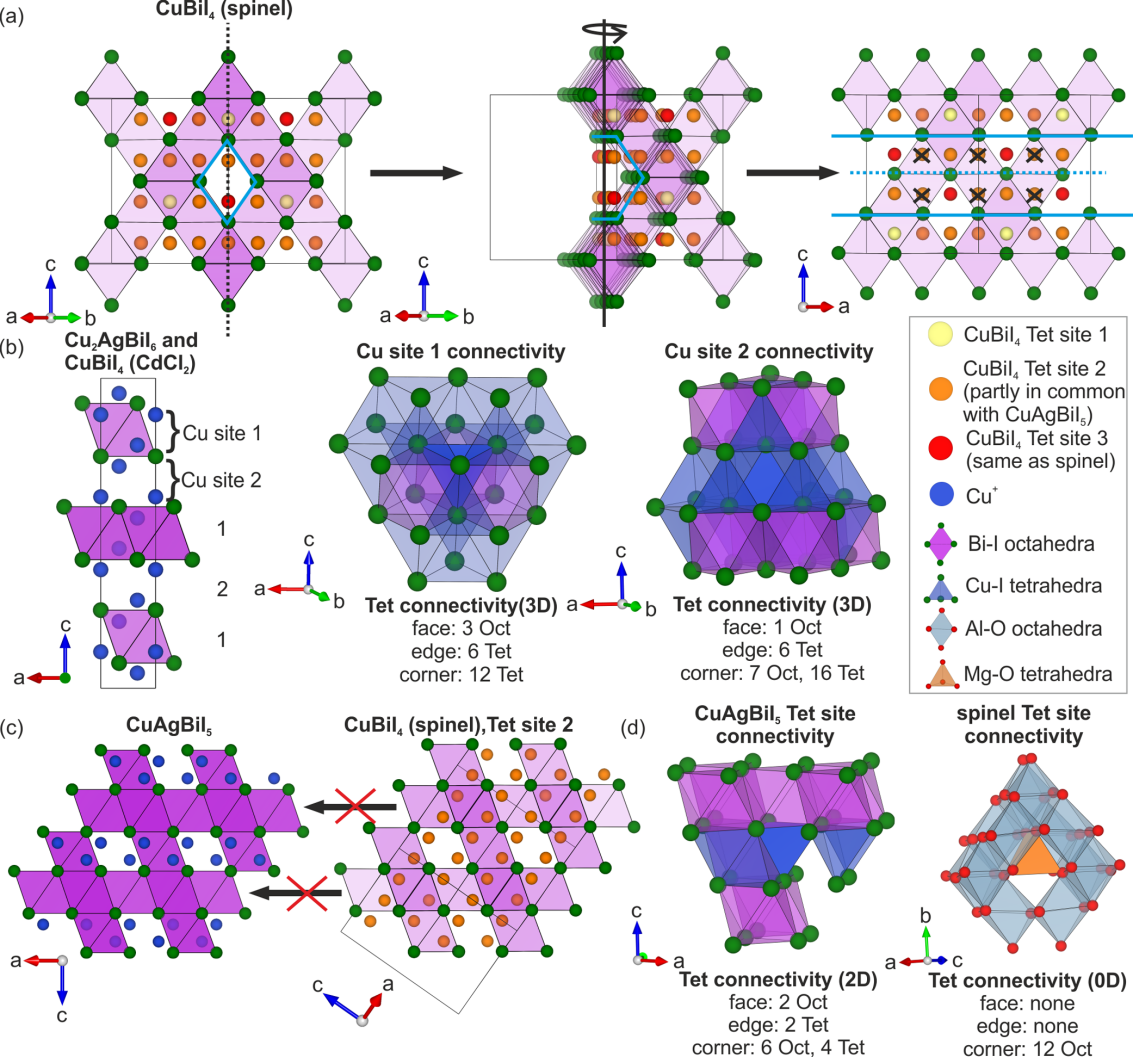
(a) The three Cu^+^ sites in CuBiI_4_ (spinel) color coded as yellow (site 1), orange (site 2), and red (site 3).^22^ A channel in the spinel octahedral (Oct) motif is highlighted in light blue, which we take a cross section of to show the tetrahedral (Tet) sites inside (sites located behind the channel, which appear to be inside due to the 2D representation of the 3D structure, have been crossed out.). The red site (site 3) is the same in spinel.^28^ Some, but not all, of the orange sites (site 2) are occupied in CuAgBiI_5_. The Tet sites in CuAgBiI_5_ can be considered as a reflection of the spinel sites with the mirror plane down the center of the channel (blue dashed line). (b) The two Cu^+^ sites in the small trigonal unit cell (Cu_2_AgBiI_6_ and CuBiI_4_ (CdCl_2_)) showing layered ordering.^2^ Also shown are the connectivities of the Tet sites, which give a 3D Tet network. (c) The layered ordering of Cu^+^ sites in CuAgBiI_5_ means they are only in layers with  Oct interstitial occupancy and do not occupy all the sites associated with tetrahedral site 2 in CuBiI_4_ (spinel). (d) The connectivity of tetrahedra in CuAgBiI_5_ and spinel, which give 2D and 0D Tet networks, respectively.

The reported compounds in the CuI–AgI–BiI_3_ phase space consist of Cu^+^, Ag^+^, and Bi^3+^ cations occupying interstitial octahedral (Oct) or tetrahedral (Tet) sites in close-packed iodide sublattices (see Figure 3). In CuI, the iodide sublattice is cubic close-packed (CCP) consisting of *ABCABC* stacking (Figure S10). In the other binaries, AgI and BiI_3_, the iodide sublattice is hexagonal close-packed (HCP) with *ABAB* stacking. Close-packed anion sublattices have 2 interstitial Tet sites and 1 interstitial Oct site per anion. For example for BiI_3_, in which the Bi^3+^ is octahedrally coordinated, the composition requires that  of Oct interstitial sites are fully occupied by Bi^3+^. BiI_3_ has layered ordering consisting of a layer with  Oct interstitial sites occupied separated by vacant layers maintaining the overall  of Oct interstitial sites occupied (Figure S11). For the room temperature CuI and AgI phases, in which the cations are tetrahedrally coordinated,  of the Tet interstitial sites are occupied giving a 3D network of corner-sharing tetrahedra. This concept can be extended to the ternary and quaternary compounds but with the added complexities of disorder and nonstoichiometric compositions. In these compounds, the cation site occupancies are below 1. The reported crystal structures show that Ag^+^, Bi^3+^, or vacancies can be found on the Oct sites, and Cu^+^ or vacancies can be found on the Tet sites. Unlike in room temperature AgI, Ag^+^ is octahedrally coordinated in the ternary and quaternary compounds. Comparing the I–I distances in the binary and ternary compounds suggests that Ag^+^ is tetrahedrally coordinated in iodide sublattices with larger I–I distances of ∼4.6 Å, such as in AgI, but is octahedrally coordinated for shorter I–I distances of ∼4.3 Å reported for the ternary and quaternary systems. Atomic disorder means that we are no longer restricted to integer ratios between the occupancy of the ions; however, we have chosen to represent nonstoichiometric compounds with a close stoichiometric composition for ease and report the measured or refined composition elsewhere in the text. It should be noted that, when we solve the structures of these systems using diffraction data, it is required that the I^–^ anion is considered to be ordered with an atomic occupancy of 1. We also normalize the measured and refined compositions to an integer amount of iodine. Here, we unravel the complex reported crystal structures of the reported ternary and quaternary compounds by breaking them down into three parts: the Oct motifs (Figure 2), the unit cells (Figure 3), and the location of the Tet interstitials occupied by Cu^+^ (Figure 4). We use our introduced nomenclature to describe CuAgBiI_5_ and summarize all compounds in Table 1.

**Table 1:** Summary of the Structural Features of the Binary, Ternary, and Quaternary Compounds from the CuI–AgI–BiI_3_ Phase Space^a^

system	close stoichiometric composition	unit cell (Figure 3)	space group	octahedral motif (Figure 2)	tetrahedral sites (Figure 4)	iodide sublattice	structure type	refs
binary	BiI_3_	trigonal	*R*3	BiI_3_-type (2D)	none	HCP	BiI_3_	(17)
	CuI	cubic	*F*43*m*	none	zinc blende (3D)	CCP	zinc blende	(15)
	AgI	hexagonal	*P*6_3_*mc*	none	wurtzite (3D)	HCP	wurtzite	(16)
ternary	Ag_1–3*x*_Bi_1+*x*_I_4_*x* < 0 (Ag-rich)	small trigonal	*R*3*m*	NaVO_2_ (3D)	none	CCP	NaVO_2_	(1, 18−21, 27)
	Ag_1–3*x*_Bi_1+x_I_4_*x* ≥ 0 (Bi-rich)	cubic	*Fd*3*m*	spinel (3D)	none	CCP	defect spinel	(1, 18−21)
	Ag_1–3*x*_Bi_1+*x*_I_4_*x* ≥ 0 (Bi-rich)	small trigonal	*R*3*m*	CdCl_2_ (2D)	none	CCP	CdCl_2_	(1)
	CuBiI_4_	small trigonal	*R*3*m*	CdCl_2_ (2D)	antifluorite (3D, layered ordering)	CCP	CuBiI_4_ (CdCl_2_)	this work
	CuBiI_4_	cubic	*Fd*3*m*	spinel (3D)	antifluorite (3D)	CCP	CuBiI_4_ (spinel)	(22)
quaternary	CuAgBiI_5_	large trigonal	*R*3*m*	spinel (3D)	CuAgBiI_5_ (2D)	CCP	CuAgBiI_5_	this work
	Cu_2_AgBiI_6_	small trigonal	*R*3*m*	CdCl_2_ (2D)	antifluorite (3D, layered ordering)	CCP	Cu_2_AgBiI_6_	(2)

a The nomenclature referred to is described in the main text and corresponding figures. Previously reported Cu_2_BiI_5_ has been omitted from the table, as its structure has not been solved.

For the CuI–AgI–BiI_3_ family of materials, the Ag^+^ and Bi^3+^ iodide octahedra are edge-sharing, as opposed to corner-sharing in the hybrid lead perovskites. Thus far, three different Oct motifs have been reported, corresponding to those found in spinel,^28^ CdCl_2_,^29^ and NaVO_2_ (Figure S12).^30^ The spinel Oct motif has been reported for AgBiI_4_, AgBi_2_I_7_, Ag_2_Bi_3_I_11_, and CuBiI_4_.^1,18,19,22^ In spinel,  of the Oct interstitial sites are fully occupied to make a 3D network shown in Figure 2a,^28^ as they are for the composition AgBiI_4_, for which Ag^+^ and Bi^3+^ equally occupy the Oct site, and there are no vacancies. When the compositions are Bi-rich (AgBi_2_I_7_, Ag_2_Bi_3_I_11_), the materials start to exhibit vacancies on these Oct sites. This is because for each Bi^3+^ added, three times as much Ag^+^ is removed for charge balance, which reduces the total Oct site occupancy to less than 50%, according to the formula Ag_1–3*x*_Bi_1+*x*_I_4_. Therefore, we refer to this Oct motif as spinel but highlight the fact that it can exhibit vacancies. CuBiI_4_ also has the spinel Oct motif, with octahedra occupied by 50% Bi^3+^ and 50% vacant, with a total Oct site occupancy of . An alternative description of the spinel Oct motif is to consider it as a vacancy-ordered rock salt in which the occupied octahedra interstitial sites are arranged in the spinel motif. Structures with the spinel Oct motif have been reported in cubic unit cells and crystallize in space group *Fd*3*m* (Figure 3a). For AgBiI_4_, there is an alternative structural description that fits single crystal and powder diffraction data sets.^1^ This alternative structural description consists of twinning of a CdCl_2_ Oct motif (Figure 2b). Like the spinel motif, the CdCl_2_ Oct motif has  of Oct interstitial sites occupied. The occupied Oct sites share edges to form 2D layers which are separated by vacant layers. In the AgBiI_4_ structure with the CdCl_2_ Oct motif, the Oct sites are occupied by 50% Ag^+^ and 50% Bi^3+^, whereas in Cu_2_AgBiI_6_, which also has the CdCl_2_ Oct motif, the octahedra are occupied by 34.6% Ag^+^ and 30.6% Bi^3+^ and are 34.8% vacant. To see if the ambiguity between the spinel and CdCl_2_ Oct motif can exist for the CuBiI_4_ powder, we fit the PXRD pattern with both. In the SI (Figure S9, Tables S1–S3), we show that diffraction data sets collected on the CuBiI_4_ powder can be alternatively fitted to a structure with a CdCl_2_ Oct motif. It is likely that compositions AgBi_2_I_7_ and Ag_2_Bi_3_I_11_ can also be represented by a twinning of structures with the CdCl_2_ Oct motif, although this has yet to be shown. Therefore, in our discussion and Table 1, we state that AgBiI_4_, CuBiI_4_, AgBi_2_I_7_, and Ag_2_Bi_3_I_11_ can all exhibit either the spinel or CdCl_2_ Oct motifs. Structures with the CdCl_2_ Oct motif are represented in a small trigonal unit cell as shown in Figure 3b in space group *R*3*m*. For the special case where the structural ambiguity between the 3D spinel and twinning of 2D CdCl_2_ structures exist, the small trigonal unit cell must be metrically cubic with lattice parameters *a*_t_ and *c*_t_ satisfying *c*_t_/2*a*_t_ = .

In Ag-rich compositions Ag_2_BiI_5_ and Ag_3_BiI_6_, overall Oct site occupancies are over 50%, where 50% is the maximum which can be occupied by the spinel and CdCl_2_ Oct motifs.^18,19^ In these structures, the excess Ag^+^ occupies Oct interstitial sites between the layers of a CdCl_2_ Oct motif. This means that every Oct interstitial site in the CCP iodide sublattice is occupied (Figure 2c); however, in the compositions reported so far, one Oct site has a full atomic occupancy, and the other has a very low occupancy meaning the structure alternates between layers of full and almost empty octahedra. For Ag_2_BiI_5_ (refined as Ag_1.92_Bi_0.83_I_5_),^18^ the Oct site in one of the layers is full with occupancy 67% Ag^+^ and 33% Bi^3+^, and the Oct site in the neighboring layer has a low occupancy of 9.6% Ag^+^. Despite this, the Oct network must be considered 3D, as every interlayer Ag^+^ ion connects octahedra from the adjacent layers. We refer to this Oct motif as NaVO_2_ (Figure S12c). Structures with the NaVO_2_ Oct motif can be represented in a small trigonal unit cell with space group *R*3*m*, as for the CdCl_2_ motif, as shown in Figure 3b. NaVO_2_ was first reported by Rüdorff,^31^ which is why Rüdorffite has been proposed to describe this family of materials,^4^ although the specific crystal structure it refers to was reported later;^30^ however, this motif only describes the ternary Ag-rich compounds. For the Cu-containing compounds CuBiI_4_ and Cu_2_AgBiI_6_, Cu^+^ occupancy is disordered over all possible Tet interstitials giving 3D corner-sharing tetrahedral connectivity. The antifluorite structure of Li_2_O is an example of this, in which Li^+^ occupies all possible Tet interstitials in the CCP O^2–^ sublattice (Figure S12d).^32^ In Li_2_O, all of the Li^+^ sites are fully occupied, whereas in CuBiI_4_ with the spinel Oct motif, there are three Cu^+^ sites with low occupancies of 0.09, 0.12, and 0.18 (Figure 4a). In Figure 4, we show that one of the Tet sites is the same as that occupied in spinel. The extra occupied Tet interstitials in CuBiI_4_ mean it is not a spinel structure, although the Oct motif is spinel, and therefore, we refer to its structure type as CuBiI_4_ (spinel). In Cu_2_AgBiI_6_ and the CuBiI_4_ structure with the CdCl_2_ Oct motif (referred to as CuBiI_4_ (CdCl_2_)), there are two Cu^+^ sites with partial layered ordering (Figure 4b). One site is in the layer with occupied Oct interstitials, and the other site in the otherwise vacant layer. The tetrahedra are face-sharing with octahedra, edge-sharing with neighboring tetrahedra, and corner-sharing with only tetrahedra (Cu site 1), or with octahedra and tetrahedra (Cu site 2), as shown in Figure 4b. Searching for a known structure type for Cu_2_AgBiI_6_ and CuBiI_4_ (CdCl_2_), using Wyckoff positions and space group to search the ICSD and Pearson’s Crystal Data,^33,34^ yields heavily disordered lithium vanadates, in which disorder has been created by partial delithiation. In particular, Cu_2_AgBiI_6_ and CuBiI_4_ (CdCl_2_) are defect-versions of Li_0.2_V_1.16_O_2_ (Figure S12e).^27^ All of the Oct and Tet interstitial sites occupied in Cu_2_AgBiI_6_ and CuBiI_4_ with the CdCl_2_ Oct motif are also occupied in Li_0.2_V_1.16_O_2_. However, Li_0.2_V_1.16_O_2_ has the NaVO_2_ Oct motif where all Oct interstitials are occupied, as opposed to the CdCl_2_ Oct motif of Cu_2_AgBiI_6_ and CuBiI_4_ (CdCl_2_) where only half of these Oct interstitial sites are occupied. This demonstrates that the partial control of ordering can give different long-range average structures, all of which consist of an arrangement of cations between layers of fully anionic close-packed layers which makes them distinct from perovskite.^1^

## CuAgBiI_5_ Crystal Structure

**Figure 5:**
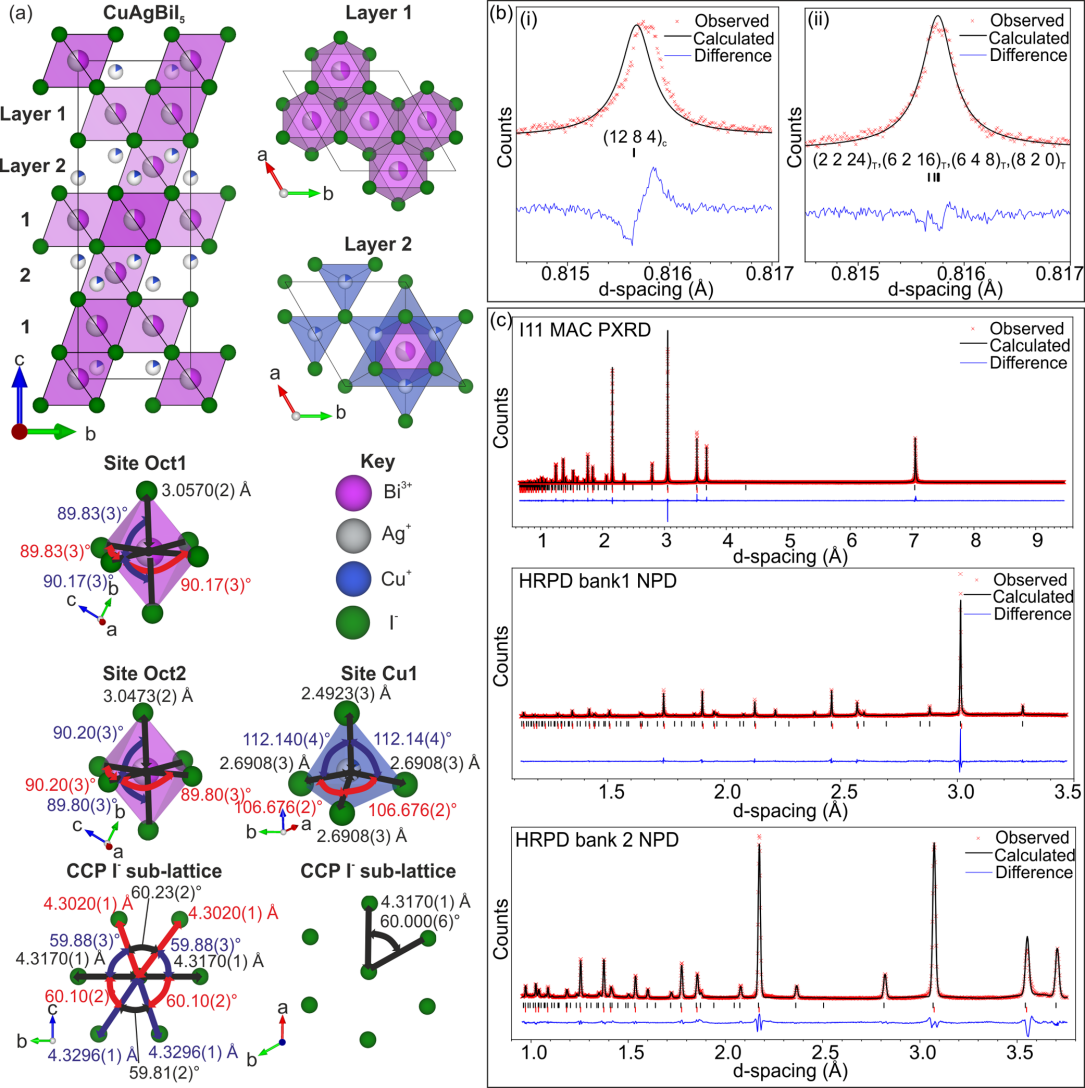
(a) The room temperature crystal structure of CuAgBiI_5_ from Rietveld refinement of combined PXRD and NPD data sets, including coordination environments. (b) A low *d*-spacing region of the fit, showing that a trigonal cell (ii) fits better than a cubic cell (i). (c) The fits to high-resolution synchrotron PXRD (MAC detector, I11, Diamond Light Source, Oxfordshire, UK) and high-resolution NPD (banks 1 and 2, HRPD, ISIS Neutron and Muon Source, Oxfordshire, UK) data sets. The goodness-of-fit parameters are presented in Table S5.

Here, we explain how we solved the crystal structure of CuAgBiI_5_ and describe the structural relationships to the known ternary and quaternary compounds. The final room temperature structure is shown in Figure 5, crystallographic information can be found in Table 2, bond distances and angles are reported in Table S4, and goodness of fit parameters of the Rietveld fits are reported in Table S5. We explore the CuI–AgI–BiI_3_ phase space and optimize synthetic parameters to isolate pure powder samples of CuAgBiI_5_ as described in the SI. In the SI, we describe in detail the exploratory synthesis and how compositional inhomogeneity has been overcome, as a useful guide for other researchers wanting to synthesize these materials. Multiple 0.25 g batches of powder are combined to form a sample massive enough for neutron powder diffraction measurements (Figure S13). Small crystals are picked out of the final powder (Figure S14) which are much more suitable for single crystal X-ray diffraction (SCXRD) than large crystals grown via chemical vapor transport (CVT). The CuAgBiI_5_ structure (Figure 5) is solved by Rietveld refinement of complementary combined room temperature high-resolution synchrotron powder X-ray diffraction (PXRD) (MAC detector, I11, Diamond Light Source, Oxfordshire, UK) and high-resolution neutron powder diffraction (NPD) (HRPD, ISIS Neutron and Muon Source, Oxfordshire, UK) data sets, with information also gathered from SCXRD data collected at 100 K.

**Table 2:** Refined Room Temperature Structural Parameters of CuAgBiI_5_

composition	refined parameters		site	atom	*x*	*y*	*z*	occupancy	*U* (Å^2^ × 10^3^)	Wyckoff position	point group (Hermann-Mauguin)
CuAgBiI_5_	formula sum	Cu3.11 Ag4.97 Bi5.05 I24	I1	I	1/2	1/2	0.25040(3)	1	21.6(1)	18h	m
	*Z*	1	I2	I	0	0	0.25040(3)	1	21.6(1)	6c	3m
	formula weight (g/mol)	4834.67	Oct1	Bi	1/2	1/2	1/2	0.421(1)	38.2(2)	9d	2/m
	crystal system	trigonal		Ag	1/2	1/2	1/2	0.414(2)	38.2(2)	9d	2/m
	space group	*R*3*m* (166)	Oct2	Bi	2/3	1/3	1/3	0.421(1)	38.2(2)	3a	3m
	cell parameters (Å)	*a* = 8.63390(5) *c* = 21.1398(2)		Ag	2/3	1/3	1/3	0.414(2)	38.2(2)	3a	3m
	cell volume (Å^3^)	1364.74(10)	Cu1	Cu	5/6	2/3	0.298(2)	0.173(2)	73(3)	18h	m
	calcd density (g/cm^3^)	5.88292									

Attempts to solve the structure from SCXRD yield a structure with the spinel Oct motif in a cubic unit cell. Unlike for AgBiI_4_, the structure cannot be alternatively fitted with a twinning of structures with the 2D CdCl_2_ Oct motif without a large negative peak in the residual electron density. Ultimately, however, the structure cannot be solved by SCXRD because trying to refine Cu^+^ sites causes an unstable refinement. This is likely due to correlation between the disordered occupancies and thermal parameters of the two Cu^+^ sites when using a single diffraction data set. Therefore, we take the confirmation of the spinel Oct motif and apply it to the combined PXRD and NPD data sets. First, the high-resolution PXRD data show that the unit cell is not cubic at lower *d*-spacings, whereas Cu–Ag–Bi–I materials with spinel Oct motifs have only been reported in cubic unit cells thus far. To account for this, we transform a structure with the spinel Oct motif in a cubic unit cell (space group *Fd*3*m*, lattice parameter *a*_c_, volume *V*_c_) to an equivalent spinel Oct motif in a trigonal cell (space group *R*3*m*, lattice parameters *a*_T_ and *c*_T_, and volume *V*_T_) using the transformation matrix shown in Figure 2a. This trigonal cell is smaller in volume than the cubic cell (*V*_T_ = 3*V*_c_/4), and it is four times larger than the trigonal cell associated with the CdCl_2_ and NaVO_2_ Oct motifs (lattice parameters *a*_t_ and *c*_t_ and volume *V*_t_) due to doubling of the *a* and *b* directions (*V*_T_ = 4*V*_t_). We therefore refer to the trigonal cell with the spinel Oct motif as the large trigonal cell and subscript lattice parameters and unit cell volumes with a capital “T” and the small trigonal cell and lattice parameters and unit cell volumes with subscripts of lowercase “t”. The unit cell relationships are shown in Figure 3. The lattice parameters of CuAgBiI_5_ refine to *a*_T_ = 8.63390(5) Å and *c*_T_ = 21.1398(2) Å. The large trigonal cell of CuAgBiI_5_ allows us to refine rhombohedral strain, defined as how far the value of *c*_T_/*a*_T_ is from  (), to a small value of 0.0132(2)%. This provides a much better fit to the PXRD data (Figure 5bii compared to 5bi). This transformation and lowering of the symmetry from cubic (space group *Fd*3*m*) to trigonal (*R*3*m*) create two Oct sites that make up the spinel Oct motif as shown in Figure 2a. The atomic occupancies of these two Oct sites must be kept equal in the Rietveld refinement, to prevent additional peaks from appearing in the calculated PXRD pattern. The occupancy of the two Oct sites Oct1 and Oct2 refine to 42.1(1)% Bi^3+^ and 41.4(2)% Ag^+^ leaving a vacancy of 16.5(2)%. Transforming a spinel Oct motif into a trigonal cell (Figure 2a) also highlights a more precise description of the spinel motif as alternating layers of  and  Oct interstitial occupancy. This cell and symmetry mean there are two different interstitial Tet sites with layered ordering so that one site belongs in the same layer as the  Oct interstitial occupancy, and the other belongs in the layers with the  Oct interstitial occupancy (Figure 4c). The Fourier difference map showed that Cu^+^ is located only in the layers with the  Oct site occupancy with a refined occupancy of 17.3(2)%. This Cu^+^ site is not the same as the Tet site occupied in spinel as shown in Figure 4a but is located as if reflected in a mirror plane running down the center of vacant octahedral channels of the spinel, which changes their location in reference to the octahedra. The positioning of the tetrahedral Cu^+^ in CuAgBiI_5_ means it is not a spinel structure despite having a (rhombohedrally distorted) spinel Oct motif. The ordering of the Tet sites in CuAgBiI_5_ is also distinct from those in the CuBiI_4_ (spinel) structure. Figure 4c shows that only a fraction of the Cu sites of CuAgBiI_5_ are occupied in comparison to CuBiI_4_ (spinel). As we cannot find any other examples of a spinel Oct motif with Tet sites ordered in this way, we refer to the tetrahedral connectivity as CuAgBiI_5_-type and suggest CuAgBiI_5_ is a new structure type in Table 1. Tetrahedra in spinel are isolated from other tetrahedra (0D), whereas in CuAgBiI_5_, a mixture of edge- and corner-sharing gives 2D tetrahedral connectivity (Figure 4d). The refined composition Cu_0.65(1)_Ag_1.04(2)_Bi_1.05(2)_I_5.00_ is within error of the average powder composition Cu_0.88(17)_Ag_1.10(6)_Bi_0.98(8)_I_5.00(11)_ measured by TEM EDX, which shows some compositional inhomogeneity in the amount of Cu remaining from the synthesis. The chemical environment of the ions in Figure 5b shows that the Oct sites have six equal Ag/Bi–I bond lengths of 3.0570(2) Å with angles alternating between 89.83(3)° and 90.17(3)°, close to 90°. The Cu^+^ is displaced away from the center of the tetrahedron, toward the apex in the direction that points along the *c*-axis, which leads to one Cu–I bond (2.4923(2) Å) being shorter than the others (2.6908(3) Å). Significantly distorted I–Cu–I angles of 112.14(4)° and 106.676(2)° are observed. We perform X-ray photoelectron spectroscopy (XPS) on bulk samples of CuAgBiI_5_ and show in the SI (Figure S15) the fitting of Cu 2p, I 3d, Bi 4f, and Ag 3d core levels. The binding energies are associated with the species Cu^+^, Ag^+^, and Bi^3+^ iodide bonding, and no metallic species are observed.

## Relationship between Composition and Octahedral Network

**Figure 6:**
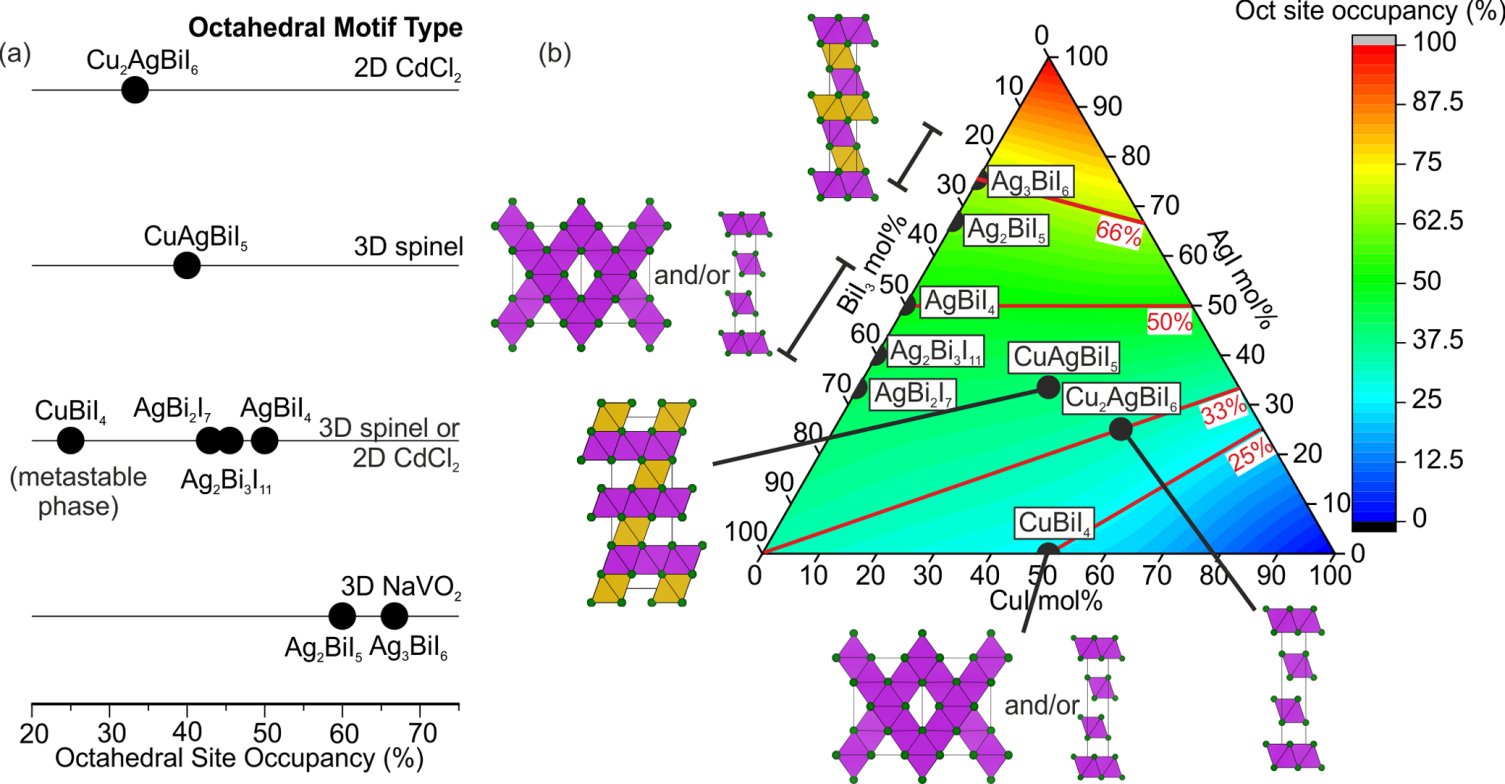
(a) The relationship between occupancy of the octahedral (Oct) sites and type of Oct motif formed, giving chemical control over dimensionality of the Oct network. CuBiI_4_ does not fit the trend but we previously found it to be metastable at room temperature.^2^ (b) The same relationship shown in the CuI–AgI–BiI_3_ phase space, where the color map and red contour lines represent total Oct site occupancy. We note that the Oct site occupancy in (b) is not representative of materials which contain tetrahedral Ag^+^ such as the room temperature structure of AgI.

The structural investigation allows suggestion of a relationship between total Oct site occupancy (composition) and the Oct motif (structure), for ternary and quaternary compositions in the CuI–AgI–BiI_3_ phase field. We note it will not be true when Ag^+^ is in tetrahedral coordination, as in room temperature AgI.^16^Figure 6 shows the type of Oct motif against the total atomic occupancies of the Oct sites for each reported ternary and quaternary compound. For high atomic Oct site occupancies above 50%, the NaVO_2_ Oct motif is obtained, which can only be obtained in the Ag-rich *x* < 0 Ag_1–3*x*_Bi_1+*x*_I_4_ compositions (Ag_2_BiI_5_, Ag_3_BiI_6_) because adding more Cu^+^ or Bi^3+^ reduces Oct site occupancy. At atomic Oct site occupancies of 50%, we have the indistinguishable 3D spinel Oct motif and/or 2D CdCl_2_ Oct motif of AgBiI_4_ and CuBiI_4_,^1^ which we also expect for the Bi-rich *x* > 0 Ag_1–3*x*_Bi_1+*x*_I_4_ compositions (Ag_2_Bi_3_I_11_, AgBi_2_I_7_). By substituting in *x* Bi^3+^ for 3*x* Ag^+^, Oct site occupancies reach as low as 45.5% and 42.9% for reported Ag_2_Bi_3_I_11_ and AgBi_2_I_7_, respectively. Continuing to decrease the Ag^+^ content will eventually lead to the BiI_3_ structure.

To obtain atomic Oct site occupancies lower than 42.9% in AgBi_2_I_7_, tetrahedral Cu^+^ is added. On the solid solution line between AgBiI_4_ and CuI, this corresponds to substituting in 4*x* Cu^+^ for every *x*(Ag^+^ + Bi^3+^) removed (Cu_4*x*_(AgBi)_1–*x*_I_4_), i.e., equal amounts of octahedral Ag^+^ and Bi^3+^ are removed. For CuAgBiI_5_, with an atomic Oct site occupancy of 40%, the 3D rhombohedrally distorted spinel Oct motif is obtained. This suggests that the spinel Oct motif can exist for total Oct site occupancies lower than 50%; however, the reduced Oct site induces a rhombohedral strain away from cubic. We note that this is the first unambiguously 3D Oct network due to the structural ambiguity for AgBiI_4_,^1^ CuBiI_4_, and also likely for Ag_2_Bi_3_I_11_ and AgBi_2_I_7_. For Cu_2_AgBiI_6_, with a total Oct site occupancy of 33%, the CdCl_2_ Oct motif is obtained showing that at some total Oct site occupancy between 40% and 33% the Oct motif becomes 2D. The outlier in the proposed relationship between total Oct site occupancy and Oct motif is CuBiI_4_, which has either the 3D spinel or twinned 2D CdCl_2_ Oct motif, with an atomic Oct site occupancy of only 25%. However, the unstable nature of the phase compared to a mixture of CuI and BiI_3_ at room temperature can be associated with the outlier Oct site occupancy.^2^ This means that CuAgBiI_5_ and Cu_2_AgBiI_6_ are examples of phase stable Cu-containing compounds attained by increasing total Oct site occupancy compared to CuBiI_4_.

## Properties of 3D CuAgBiI_5_ Compared to 2D Cu_2_AgBiI_6_

The distinct 3D and 2D Oct motifs of CuAgBiI_5_ and Cu_2_AgBiI_6_, respectively, allow us to investigate structure–property relationships. To see if there is any difference in stability, we seal CuAgBiI_5_ and Cu_2_AgBiI_6_ powders in capillaries of laboratory air, dry synthetic air, and helium atmospheres and expose them to the solar spectrum for varying amounts of time. Measuring the stability of the powders enables us to probe the stability of the compound without solution processed induced defects, such as surface effects and grain boundaries present in thin films. After 1 week in the solar spectrum, we see that CuAgBiI_5_ begins to change color from dark red to yellow on the side that is irradiated. This does not correspond to any changes in the PXRD patterns (Figure S16), but we do observe an extra peak in the Raman Spectra (Figure S17). This change occurs under all atmospheres and does not happen in control samples kept in the dark and air, meaning it is a light induced change. In contrast, we do not see any sign of decomposition in Cu_2_AgBiI_6_. This may be indicative of increased phase stability of the 2D CdCl_2_ Oct motif of Cu_2_AgBiI_6_ compared to the strained CuAgBiI_5_ 3D spinel Oct motif. It is unlikely to be due to the reduction of photosensitive Ag–I bonds in Cu_2_AgBiI_6_ compared to CuAgBiI_5_, because the more Ag-rich AgBiI_4_ exposed to the same conditions did not show this decomposition.^2^ Although we report this instability of CuAgBiI_5_, it does not necessarily mean that 3D Oct networks are intrinsically less stable than the 2D Oct networks, and the instability may not persist in related systems via chemical substitution. Furthermore, it should be highlighted that thermodynamically stable compositions quenched from the synthesis temperature of 350 °C (CuAgBiI_5_ and Cu_2_AgBiI_6_) are not necessarily the most thermodynamically stable compositions that would be obtained via low temperature solution-processing techniques or at photovoltaic device operating temperatures.

To characterize the optoelectronic properties of CuAgBiI_5_, we solution process films as described in the SI. It is particularly challenging to dissolve the powders in solutions concentrated enough to form films with high coverage, to prevent powder precipitating during deposition (which causes phase segregation), and to obtain a smooth, shiny surface. These challenges are overcome by using a mixed DMSO/pyridine solution, depositing from hot solutions onto a preheated substrate, and using a two-step annealing procedure, respectively. We fit the XRD pattern of thin films deposited on microscope slides to a large trigonal cell in the *R*3*m* space group with lattice parameters *a* = 8.724(1) Å and *c* = 20.800(5) Å (Figure S18a). The *a* and *c* parameters of the thin films are significantly larger and smaller than those of the CuAgBiI_5_ powder, respectively. To investigate this, we measure the composition of the films by SEM EDX and find them to have an average composition of Cu_0.82(5)_Ag_0.96(9)_Bi_1.07(4)_I_3.98(13)_ (Figure S18b) and significantly different metal ratios compared to Cu_2.52(9)_Ag_1.02(7)_Bi_0.82(11)_I_6.00(20)_ measured for Cu_2_AgBiI_6_ thin films by TEM EDX.^2^ While the composition of the metal cations is within 1σ of those measured for the powder, there is a large iodine deficiency of 20(3)%. It is not clear at which stage in the process the iodine is lost, and further optimization of film deposition will look to rectify this. The films show a certain level of roughness which can be seen in Figure S18c, as the film differs from a perfectly shiny black reflective surface, which arises from the morphology we show in the SEM images in Figures S18d and e; however, the films were of sufficiently high quality for spectroscopic analysis.

**Figure 7:**
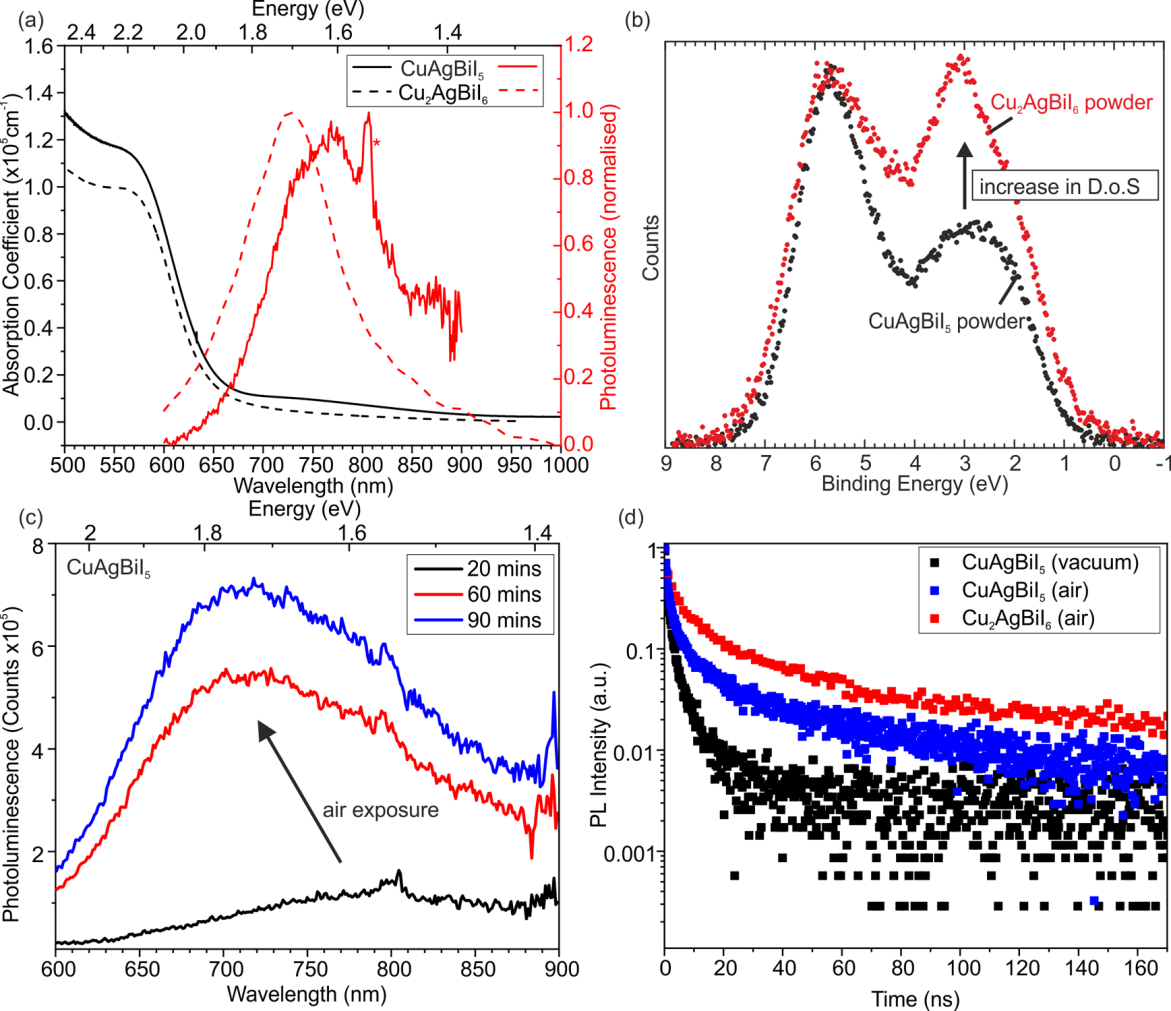
Absorption coefficient and PL measured on CuAgBiI_5_ (solid lines) and Cu_2_AgBiI_6_ (dashed line) thin films. The data for Cu_2_AgBiI_6_ is taken from Sansom et al.^2^ The PL spectra of CuAgBiI_5_ and Cu_2_AgBiI_6_ were measured in vacuum and air, respectively. (b) The density of states of the valence band measured on CuAgBiI_5_ (black) and Cu_2_AgBiI_6_ (red) powders, measured by XPS. (c) The shift and increase in the PL signal of CuAgBiI_5_ thin films exposed to air. (d) TRPL of CuAgBiI_5_ thin films measured in vacuum (black) and air (blue), compared to Cu_2_AgBiI_6_, measured in air (red).

Optical properties of CuAgBiI_5_ thin films are measured to see how the 3D spinel Oct motif of CuAgBiI_5_ compares with the 2D CdCl_2_ Oct motif in Cu_2_AgBiI_6_. Surprisingly, the absorption spectra of CuAgBiI_5_ and Cu_2_AgBiI_6_ are very similar, therefore consisting of a very similar band gap and absorption coefficients (Figure 7a). The optical absorption spectra measured for CuAgBiI_5_ deposited on z-cut quartz substrates show a clear onset at approximately 680 nm (1.8 eV), similar to the previously reported Cu_2_AgBiI_6_,^2^ rising to a value of over 1 × 10^5^ cm^–1^ for the absorption coefficient just above the band gap. A rough estimate of the band gap can be obtained from the inflection point of the onset of the absorption coefficient, and this gives a value of 2.02 eV, again similar to the value of the band gap reported for Cu_2_AgBiI_6_ (2.06 eV). This shows that lowering of the dimensionality of the Oct motif does not increase the band gap and charge confinement like it does for the corner-sharing networks of perovskites. We perform XPS on CuAgBiI_5_ and Cu_2_AgBiI_6_ powders to investigate the density of states at the top of the valence band (Figure 7b). We see an increase in density of states at the top of the valence band for Cu_2_AgBiI_6_ compared to CuAgBiI_5_, which, based on the composition, backs up the theoretical calculations for Cu_2_AgBiI_6_ suggesting Cu 3d states at the top of the valence band.^2^ In the SI, we show the energies of the valence and conduction bands with respect to vacuum for CuAgBiI_5_ compared to Cu_2_AgBiI_6_ (Figure S19). We point out that it is plotted by using the ionization potential measured on bulk samples, due to the surface sensitivity of solution-proceed thin films, and the optical band gap measured on thin films, due to the diffuse reflectance of powder samples broadening the optical absorption edge. The data suggest that both the valence and conduction bands of CuAgBiI_5_ (5.47 and 3.45 eV, respectively) are slightly lower in energy than Cu_2_AgBiI_6_ (5.21 and 3.15 eV, respectively). The lower valence band position may be due to the decreased amount of Cu^+^. The inclusion of Cu^+^ in the band edge states suggests a functionalization which contrasts with 2D perovskites; for example, calculations show that in *A*_2_Pb*X*_4_ compounds, in which *A* cations separate layers of Pb–I octahedra, both inorganic and organic *A* cations have been shown not to contribute to band-edge states.^35,36^ The lower conduction band, theorized to be dominated by Bi 6p and I 5p states,^2^ may be due to the increased connectivity of the Oct network. The shift in the conduction band position of 0.3 eV is relatively small compared to the shift of 1.63 eV observed between the 3D MAPbI_3_ and 2D BA_2_PbI_4_ end members in the Ruddlesden–Popper (BA)_2_(MA)_*n*−1_Pb_*n*_I_3*n*+1_ series.^37^ This lower sensitivity of the Cu–Ag–Bi–I materials toward the dimensionality of the octahedral network may be due to the presence of the uninterrupted CCP iodide sublattice which maximizes band dispersion and helps maintain electronic connectivity. We also note the presence of some subgap absorption between approximately 950–700 nm, as measured by Fourier transform infrared (FTIR) spectroscopy, which is possibly due to subgap defect states similar to those observed by Photo-Thermal Deflection Spectroscopy (PDS) in Cu_2_AgBiI_6_, although we cannot rule out scattering of long-wavelength light from the rough film surfaces leading to lower light transmission in this region but still allowing for the red transmitted light observed when the films are backlit (Figure S7f).

We measure steady-state photoluminescence (PL) spectra on the same thin film under continuous wave excitation in vacuum, with CuAgBiI_5_ showing weak, broad emission peaking at approximately 760 nm (1.63 eV), giving a Stokes shift of 390 meV, similar to, though slightly larger than, that observed in Cu_2_AgBiI_6_ and characteristic of emission from localized charge-carrier states, which has been proposed as the source of PL emission in Cu_2_AgBiI_6_,^25^ as well as for Cs_2_AgBiBr_6_ (Figure 7a).^6,38,39^ Compared to Cu_2_AgBiI_6_, the PL peak of CuAgBiI_5_ is slightly shifted to lower energies (1.71 eV for Cu_2_AgBiI_6_) with a slightly higher Stokes shift (350 meV for Cu_2_AgBiI_6_). The PL peak of the CuAgBiI_5_ thin film is fitted to a Gaussian peak shape with an fwhm of 312(6) meV, slightly wider than the fwhm of 289 meV extracted for Cu_2_AgBiI_6_ films. The sharp PL peak at 800 nm marked by an asterisk is from the second diffraction of the excitation laser signal from the diffraction grating in the detection setup. The PL signal for ternary Ag–Bi–I compounds is rarely reported due to it being too low in intensity to measure, but as the intensity seems more intense for the quaternary Cu-containing compounds, we took the chance to measure it in some detail. To understand the potential impact of atmospheric and light-induced effects on CuAgBiI_5_, a fresh sample is left in air and darkness for 90 min, during which steady-state PL spectra are measured after 20, 60, and 90 min (Figure 7c). The thin film is only illuminated for very brief (ca. 15 s) periods during the PL measurements, during which acquisitions are taken every 3 s, after 20, 60, and 90 min, respectively. The results show PL spectra after 20 min that are similar to those measured on fresh films in vacuum but which subsequently display a clear blue-shift and large rise in PL intensity under prolonged exposure to air. A similar variation of PL spectra with atmosphere has been widely reported for conventional metal-halide perovskites.^9,40−43^ Exposure to air has been observed to lead to significant increases in PL intensity across lead-iodide and -bromide perovskites, and this behavior has been ascribed to the passivation of defects by oxygen.^40,42,43^ The behavior observed here for CuAgBiI_5_ films in air could follow a similar process, where deeper trap states are passivated by oxygen over time, deactivating nonradiative recombination pathways and leading to higher-energy emission and an increase in the PL intensity. This is confirmed by transient decays measured on a film after exposure to air (Figure 7c) for which the decays show a slightly stronger fluence dependence (Figure S21), with longer lifetimes at lower fluences, and a stretched exponential fit to the lowest-fluence decay gives an average lifetime of *τ*_*av*_ = 17.9 ns, much longer than for the fresh sample measured in vacuum, and approaching the values fitted for Cu_2_AgBiI_6_ in air (*τ*_*av*_ = 33 ns). This suggests that the differences seen between the PL peak position and intensity of CuAgBiI_5_ (measured in vacuum) and Cu_2_AgBiI_6_ (measured in air) can be accounted for by defects induced by film processing routes and exposure to atmosphere before and during measurements, rather than intrinsic material properties.

To determine whether the observed changes in CuAgBiI_5_ PL over time are caused by light-induced effects, PL spectra are recorded at 3 s intervals under continuous illumination by the laser after 20 and 90 min for one sample (Figure S20). When we measure under constant illumination the spectra of CuAgBiI_5_, it shows a drop in intensity but no change in spectral shape, a process sometimes described as “photodarkening”, implying that light-induced effects are not the source of the blue-shift of the spectrum and increase in PL intensity. Photodarkening has been observed in lead-halide perovskites, under both vacuum and nitrogen,^40,41^ which in one case has been ascribed to an increased density of hole traps forming under constant illumination.^43^ However, in our case, the very high laser excitation intensity is required to measure PL spectra, of approximately 40 Wcm^–2^, suggesting that the observed decrease in intensity is likely due to the degradation of the sample region under illumination leading to the creation of point defects. This is supported by the observation of small burn marks on the thin films at the end of the PL measurements. This shows that the atmosphere and light exposure of the sample before and during measurements should be carefully chosen and detailed when reporting PL spectra.

To gain an insight into the charge-carrier lifetimes in CuAgBiI_5_, we carry out time-resolved PL measurements in vacuum using Time-Correlated Single Photon Counting (TCSPC, see the SI for details). The transient decay shown in Figure 7d of CuAgBiI_5_ measured in vacuum on a fresh sample shows a very fast initial decay, on the order of 1 ns with no fluence dependence (Figure S21) to the decays across 3 orders of magnitude. The lowest-fluence decay is fitted with a stretched exponential, yielding an average lifetime of *τ*_*av*_ = 0.73 ns and a stretching exponent of β = 0.32.^44^ The low value of β is indicative of a highly heterogeneous decay, very similar to that observed in both Cs_2_AgBiBr_6_ and Cu_2_AgBiI_6_,^2,5^ and is likely due to a distribution of trap states with slightly varying trapping dynamics. The very short lifetime and lack of fluence dependence of the decays are indicative of a high trap density in the CuAgBiI_5_ films, leading to fast trap-mediated recombination and scarcity of radiative band-to-band recombination, consistent with the weak steady-state PL emission. This finding is further supported by time-resolved emission spectra, also measured in vacuum using TCSPC on a fresh sample and shown in Figure S21. We also observe evidence of a high-energy emission, less Stokes-shifted around 600 nm over the first 1 ns, where the emission band at 600 nm decreases in intensity relative to the main peak, and the transient decay at 600 nm is faster than that at 720 nm.

Finally, optical-pump terahertz-probe spectroscopy is used to measure the effective charge-carrier mobility for two thin films of CuAgBiI_5_, yielding values of 1.7(2) and 1.3(2) cm^2^ V^–1^ s^–1^, as shown in Figure S22. These values are comparable to charge-carrier mobilities measured for both Cs_2_AgBiBr_6_ and Cu_2_AgBiI_6_^2,6,45^ (0.8 cm^2^ V^–1^ s^–1^ and 1.7(5) cm^2^ V^–1^ s^–1^, respectively) and are lower than values reported across conventional metal-halide perovskites.^46,47^ Charge-carrier mobility is influenced by intrinsic effects, such as scattering off of ionized impurities or couplings between charge carriers and the crystal lattice, and extrinsic effects, such as poor crystallinity and high energetic disorder or scattering off defects.^46^ Given the high trap density that is apparent from the other spectroscopic measurements, it is possible that a reduction in trap density, along with enhanced crystallinity and reduced energetic disorder, could lead to an improvement in the charge-carrier mobilities for CuAgBiI_5_, although the low values for charge-carrier mobilities^2,48,49^ and fast charge-carrier recombination^39,49^ reported across a variety of silver–bismuth compositions could be indicative of more fundamental limitations to charge-carrier transport in these materials that require further chemical tuning to improve.^50^

## Conclusion

CuAgBiI_5_ is the first compound in the CuI–AgI–BiI_3_ phase field with an unambiguously 3D spinel Oct motif. The 3D Oct network has been obtained via chemical tuning, namely the total occupancy of the Ag^+^ and Bi^3+^ Oct sites, which allows selectivity between a spinel (3D), CdCl_2_ (2D), or NaVO_2_ (3D but with layered ordering) type Oct motif. We find no significant changes in band gap, absorption coefficient, PL, PL lifetimes, charge-carrier mobilities, and charge-carrier confinement (the presence of large excitonic peaks in the absorption coefficient) between the 3D Oct network of CuAgBiI_5_ and the 2D Oct network of Cu_2_AgBiI_6_. This could be due to the close-packed iodide sublattices or presence of tetrahedral Cu^+^ sites at the top of the valence band which provide enhanced electronic connectivity, thus mitigating against any changes to the electronic states due to the reduction in dimensionality of the Oct network. This contrasts with the 2D perovskites in which the cations separating the layers of Pb–I octahedra do not contribute to band-edge states. Therefore, the optoelectronic properties of Cu–Ag–Bi–I materials have a lower sensitivity toward the dimensionality of the Oct network compared to the lead halide perovskites, and thus useful materials are not restricted to 3D Oct networks. CuAgBiI_5_ shows a light-induced change in color and Raman spectra when exposed to the solar spectrum even under inert atmosphere, indicating that Cu_2_AgBiI_6_ may be the preferable composition with regards to long-term stability. We note that substituting Ag^+^ and Bi^3+^ for other cation pairings in the future may change the reliance of the optoelectronic properties on the dimensionality of the network. Structural understanding and initiating the discussion of composition–structure–property relationships will allow the materials community to envisage ways to further improve properties with the goal of applying these materials, and related materials via chemical substitution, into useful optoelectronic devices. Beyond materials improvement, there is future scope for advances resulting from optimization of processing, passivation, and device architectures.

## Supporting Information Available

Sample preparation, characterization methods, and exploratory synthesis details (Figures S1–S8), figures to support main text (Figures S9–S22, Tables S1–S3), and CuAgBiI_5_ structural and refinement parameters (Tables S4 and S5) (PDF)

## Accession Codes

CCDC 2107462–2107465 contain the supplementary crystallographic data for this paper. These data can be obtained free of charge via www.ccdc.cam.ac.uk/data_request/cif, or by emailing data_request@ccdc.cam.ac.uk, or by contacting The Cambridge Crystallographic Data Centre, 12 Union Road, Cambridge CB2 1EZ, UK; fax: +44 1223 336033.

## Notes

The data as presented in this paper is freely available at https://datacat.liverpool.ac.uk/id/eprint/1439. The DOI is 10.17638/datacat.liverpool.ac.uk/1439.
