# Supplementary material for: Chemical Control of the Dimensionality of the Octahedral Network of Solar Absorbers from the CuI–AgI–BiI3 Phase Space by Synthesis of 3D CuAgBiI5
Source: Inorg Chem. 2021 Nov 9;60(23):18154–67. doi: 10.1021/acs.inorgchem.1c02773 (PMC8653216; doi:10.1021/acs.inorgchem.1c02773)
Supplement: Supplementary file 1 — ic1c02773_si_001.pdf [file ic1c02773_si_001.pdf]

Supplementary Information

Chemical Control of the Dimensionality of the Octahedral Network of Solar Absorbers from the CuI−AgI−BiI$_3$ Phase Space by Synthesis of 3D CuAgBiI$_5$

Harry C. Sansom,[1,2] Leonardo R.V. Buizza,[2] Marco Zanella,[1] James T. Gibbon,[3] Michael J. Pitcher,[1,‡] Matthew S. Dyer,[1] Troy D. Manning,[1] Vinod R. Dhanak,[3] Laura M. Herz,[2] Henry J. Snaith,[2] John B. Claridge,[1] Matthew J. Rosseinsky[1,*]

1. University of Liverpool, Department of Chemistry, Materials Innovation Factory, 51 Oxford St. Liverpool L7 3NY, U.K.
2. University of Oxford, Clarendon Laboratory, Department of Physics, Parks Road, Oxford OX1 3PU, U.K.
3. University of Liverpool, Stephenson Institute for Renewable Energy and Department of Physics, Oxford St. Liverpool L69 7ZF, U.K.
* E-mail: M.J.Rosseinsky@liverpool.ac.uk

Contents

Sample Preparation

- Optimised $CuAgBiI_5$ Powder Synthesis S3
- $CuAgBiI_5$ Crystals S3
- $CuAgBiI_5$ Film Deposition S3

Characterisation Methods

- Powder X-ray diffraction (PXRD) S4
- Neutron powder diffraction S4
- Single crystal X-ray diffraction (SCXRD) S4
- Compositional Analysis S4
- Photostability Experiment S4
- Raman Spectroscopy S5
- X-ray Photoelectron Spectroscopy (XPS) S5
- Fourier-Transform Infrared Spectroscopy (FTIR) S5
- Steady-State Photoluminescence Measurements S5
- Time-Resolved Photoluminescence Measurements S6
- Time-Resolved Photoluminescence Fitting S6
- Optical-Pump Terahertz-Probe Spectroscopy S6
- Calculation of Charge-Carrier Mobility S6

Exploratory Synthesis – $CuAgBiI_5$ (and Cu_2AgBiI_6) discovery (Figures S1-S8) S8

$CuBiI_4$ structure with the spinel and $CdCl_2$ octahedral motifs (Figure S9, Tables S1-S3) S14

Supplementary Figures

- S10. The structures of CuI, AgI and BiI_3 S17
- S11. BiI_3 octahedral motif S18
- S12. The structures of spinel, $CdCl_2$, $NaVO_2$, antifluorite Li_2O and $Li_{0.2}V_{1.16}O_2$. S19
- S13. Screening of small batches of powder for neutron sample S20
- S14. Crystal used for SCXRD S21
- S15 X-ray photoelectron spectroscopy core levels S22
- S16. Photostability (PXRD) S23
- S17. Photostability (Raman Spectra) S24
- S18. Thin film characterisation S25
- S19. Band positions with respect to vacuum S26
- S20. PL light soaking S27
- S21. Time-resolved emission spectra S28
- S22. Mobility measurements S29

Supplementary Tables

- S4. Bond distances and bond angles for $CuAgBiI_5$ S30
- S5. Rietveld refinement parameters S31

Sample Preparation

Optimised $CuAgBiI_5$ Powder Synthesis

CuI (0.0318g, 99.999% Sigma–Aldrich), AgI (0.0653g, 99.999% Alfa Aesar) and BiI_3 (0.1530g, synthesised as previously reported)[1] powders (nominal composition $Cu_{0.68}Ag_{1.14}Bi_{1.06}I_{5.00}$, just off the $Cu_{4x}(AgBi)_{1-x}I_4$ solid solution line) were ground together in a pestle and mortar and pressed in to a pellet with a 5 mm diameter. The pellet was sealed in a fused silica tube. The fused silica tubes had a 6 mm internal diameter, 1 mm thick walls and were sealed at 10^{-4} mbar to 15 cm in length. The sample in the tube was cooled in liquid N_2 during the evacuation and sealing process to avoid sublimation of the iodides. The tube was placed upright in a furnace and heated at 5°C/min to 350°C and kept there for 5 days. The tube was taken out after 5 days and the bottom half of it (containing the pellet) was quenched to room temperature in a water bath. The tube was scored and carefully broken open to retrieve the pellet which was then ground in to a dark red powder. Direct scale up of the $CuAgBiI_5$ powder synthesis from 0.25g to 2.5g led to a small decrease in compositional homogeneity. Therefore, to produce large samples for neutron powder diffraction (NPD) experiments, 0.25g batches of $CuAgBiI_5$ were synthesised and screened for their lattice parameter, purity by PXRD, and average TEM EDX composition and then combined (Figure S13).

$CuAgBiI_5$ Crystals

A small crystal approx. 20 µm × 15 µm × 10 µm, with an average SEM EDX composition of $Cu_{0.77(1)}Ag_{1.27(3)}Bi_{1.10(5)}I_{5.00(4)}$, was picked out of the $CuAgBiI_5$ powder sample for structural studies via SCXRD (Figure S14).

$CuAgBiI_5$ Film Deposition

156.5 mg AgI and 393.1 mg BiI_3 were dissolved in 0.8 ml DMSO at 100°C over 15 minutes with constant stirring. In a separate vial, 127 mg of CuI powder were dissolved in 0.5 ml pyridine at 100°C over 15 minutes with constant stirring. The AgBiI in DMSO and CuI in pyridine solutions were quickly filtered through a 0.22 µm pore-size, 13 mm diameter PTFE filter and combined into a single vial. The solutions were kept stirring at 100°C during deposition. Glass substrates were cut to size (approx. 24mm x 24 mm) and sonicated in soap and DI water, acetone and IPA for 15 minutes, dried using an N_2 gun, then further cleaned in a U.V.-Ozone generator. After which, the substrates were quickly transferred to a dry box and heated to 150°C for deposition. 100 ul of the hot CuAgBiI solution was deposited dynamically on to the hot substrate and spun at 4000 rpm for 45 seconds on a spincoater. The films were then annealed at 50°C for 30 minutes in air, over which the films turned from a clear orange film to a dark red-brown film. The film was then annealed at 150°C for 15 seconds in air to form the dark black film. Films were transferred to, and kept in, a N_2 filled glovebox until used for measurements.

Characterisation Methods

Powder X-ray Diffraction (PXRD)

Initial compositional screening and solution processing experiments made use of PXRD data measured on a Panalytical X'Pert Pro diffractometer using Co $K\alpha_1$ radiation (λ = 1.7890 Å) in Bragg–Brentano geometry and an X'Celerator detector. Phase identification was carried out using the X'Pert HighScore Plus (Version 2.2a)[2] with the PDF-2-ICDD database. PXRD data of capillaries used for photostability assessment were measured on a Bruker D8 Advance diffractometer using monochromated Mo $K\alpha_1$ radiation (λ = 0.7093 Å). PXRD patterns used for detailed structural analysis were collected at room temperature using the MAC detectors on the I11 beamline at Diamond Light Source (RAL, Oxfordshire, U.K.) with a wavelength of λ = 0.825898 Å. Samples were mixed with 50 vol% amorphous boron to reduce absorption effects and contained within 0.3 mm diameter borosilicate capillaries. TopasAcademic (Version 5) was used to perform Pawley fittings and Rietveld refinements of the data. VESTA[3] was used for graphical representation of the structures. X-ray diffraction patterns collected on $CuAgBiI_5$ films were measured using a Panalytical X'Pert powder diffractometer, using radiation from a Cu K-α_1 source, across 2θ values ranging from 5-40°.

Neutron Powder Diffraction (NPD)

High-resolution time-of-flight (ToF) data were collected on the HRPD instrument at ISIS Neutron and Muon Source (RAL, Oxfordshire, U.K.) of powder samples at room temperature packed in to vanadium cans with a 6 mm diameter. Data from bank 1 and bank 2 were used for Rietveld refinement in combination with the PXRD I11 dataset.

Single Crystal X-ray Diffraction (SCXRD)

Data were collected at 100K on a Rigaku MicroMax-007 HF diffractometer with a molybdenum rotating anode microfocus source and a Saturn 724+ detector using Rigaku Crystal Clear v2.0. Unit-cell indexation, data integration, and reduction were performed using Rigaku CrysAlisPro v171.38.43. The structure was partially solved using SHELX-2013,[4] implemented through Olex2.[5]

Compositional Analysis

Scanning Electron Microscopy (SEM) and Energy Dispersive X-ray Spectroscopy (EDX) was used to measure the composition as a direct elemental analysis technique. Measurements were carried out using a Hitachi S-4800 SEM with an Oxford Instruments model 7200 EDS X-ray detector. Quantification was carried out using the microanalysis suite of the Inca Suite software (Version 4.15). All powders and crystals were sputtered with 15 nm Au to limit charging effects. Transmission Electron Microscopy (TEM) EDX was carried out using an JEOL JEM 2000FXII TEM microscope operating a W electron source operated at 200 kV, using an EDAX EDX detector, with quantification carried out using EDAX Genesis Spectrum (Version 5.217, 21-Jan-2008). All samples were prepared by spreading a finely ground powder onto carbon coated Au grids. Beam intensity had to be lowered, by increasing the spot size of the beam, as to not decompose the samples. All compositions calculated from EDX measurements were normalised to the nominal iodide content unless otherwise stated.

Photostability Experiment

For the experiments, a Solar Light Model 16S-300-002 Solar Simulator was used which has a spectral output that complies with air mass 1.5 (AM1.5) per the ASTM standard definition. The combination of

neutral density filters and lamp-to-sample distance allowed for the tuning of the intensity of the incident light to 1000 Wm^{-2} as measured by a Solar Light Pyranometer PMA2144 and datalogging radiometer PMA2100. Sample temperatures were monitored using a T-type thermocouple and were found to stay below 35°C. For measurements in sealed atmospheres, powder was loaded into thin-walled (0.01 mm wall thickness) borosilicate capillaries under ambient air, dry synthetic air, and helium, and these were sealed with a gas-oxygen torch. The capillaries were then placed in the solar simulator and subjected to the full solar spectrum at an intensity of1000 Wm^{-2}.

Raman Spectroscopy

The measurements were carried out on a Renishaw inVia Reflex with a Leica microscope utilising a 633 nm wavelength red laser with a maximum power of 6.5 mW. The powder samples were measured in borosilicate capillaires and were exposed to 0.5% of the maximum laser power to avoid decomposition of the sample. The spot size was 5 µm.

X-ray Photoelectron Spectroscopy (XPS) Measurements were conducted on powders using a SPECS monochromatic Al Kα (1486.6 eV) X-ray source and a PSPMCD5 analyser calibrated to the Ag $3d_{5/2}$ peak. For sample work function measurements, a 20 V bias was applied to the sample to shift the work function from the analyser work function. Samples were measured at a base pressure of 10^{-10} mbar. Further details, including spectrometer calibration, can be found elsewhere.[6] Charge neutralisation at the surface was achieved by means of a low energy electron flood gun and subsequent correction of the binding energy scale to the adventitious C 1s peak (284.8 eV).

Fourier-Transform Infrared Spectroscopy (FTIR)

Absorption spectra were taken using a Bruker Vertex 80v Fourier-Transform Infrared (FTIR) spectrometer, with a tungsten halogen source and a silicon diode detector. Measurements were carried out under low vacuum (< 5 mbar). To calculate the absorption coefficient, film thickness was measured using a Veeco Dektak 150 profilometer, which gave a thickness of 320 ± 30 nm.

Absorption coefficients were calculated as:

$$\alpha = -\frac{1}{d}\ln\left(\frac{T}{1-R}\right),$$

where d is the sample thickness, $T = \frac{t_{sample}}{t_{ref}}$ and $R = \frac{r_{sample}}{r_{ref}}$, and a blank quartz substrate and silver mirror were used as references for the transmission and reflection, respectively.

Steady-State Photoluminescence Measurements

Photoluminescence (PL) spectra were measured following excitation by a 398 nm continuous wave laser (PicoHarp, LDH-D-C-405M) at a power density of 40.5 Wcm^{-2}. The emitted PL was collected and coupled into a grating spectrometer (Princeton Instruments SP-2558), after which light was detected by an iCCD camera (PI-MAX4, Princeton Instruments).

Measurements on three fresh films were carried out under vacuum ($< 5 \times 10^{-2}$ mbar). Subsequently, two fresh films were exposed to air over 90 minutes, and short (15 s) measurements were carried out

after 20, 60 and 90 minutes at a power density of 39.0 Wcm^{-2}. The measurements involved five acquisitions being taken, every 3 s, under constant illumination over 15 s.

Time-Resolved Photoluminescence Measurements

Time-Correlated Single Photon Counting was carried out using the same laser as above to photoexcite the thin films, but in pulsed excitation mode with a repetition rate of 5 MHz at fluences of 1480, 410, 180, 50 nJcm^{-2}. Photoluminescence was collected using the same monochromator, with a photon-counting detector (PDM series from MPD). Timing is controlled electronically using a PicoHarp300 event timer. Measurements were carried out under vacuum ($< 5 \times 10^{-2}$ mbar) and air, as discussed in the main text.

Time-Resolved Photoluminescence Fitting

A least-squares fit was carried out on the highest-fluence PL decay using a stretched exponential of the form $I = I_0 e^{-\left(\frac{t}{\tau_{char}}\right)^{\beta}}$ between 0 – 150 ns, and the average decay lifetime is calculated as $\tau_{av} = \left(\frac{\tau_{char}}{\beta}\right)\Gamma\left(\frac{1}{\beta}\right)$.[7]

Optical-Pump Terahertz-Probe Spectroscopy

An amplified laser system (Spectra Physics, MaiTai - Ascend - Spitfire) with a central wavelength 800 nm, 35 fs pulse duration and 5 kHz repetition rate was used to generate THz radiation via the inverse spin hall effect[8] and was detected using free-space electro-optic sampling with a 1 mm-thick ZnTe (110) crystal, a Wollaston prism and a pair of balanced photodiodes. The THz pulse was measured in transmission geometry. The pump beam was frequency-doubled to 400 nm by a β-barium borate (BBO) crystal. Measurements were carried out under low vacuum (< 10^{-2} mbar). Charge-carrier mobilities were calculated from the initial transmitted signal at time t = 0 ps, at fluences of 4.9, 12.5, 25 $\mu\mathrm{Jcm}^{-2}$.

Calculation of Charge-Carrier Mobility

At low fluences (< 50 $\mu\mathrm{Jcm}^{-2}$) there is a linear relationship between the charge-carrier mobility μ and the change in photoconductivity of the sample ΔS:

$$\mu = \frac{\Delta S\, A_{eff}}{Ne}. \; S1$$

The change in photoconductivity can in turn be related to the fractional change in terahertz transmission using a standard formula for thin-film samples:[9]

$$\Delta S = -\epsilon_0 c(n_A + n_B)\left(\frac{\Delta T(t=0)}{T}\right). \; S2$$

We can calculate the number of photoexcited charge carriers using:

$$N = \phi \frac{E\,\lambda}{h\,c}\left(1 - R_{pump}(\lambda)\right)\left(1 - T_{pump}(\lambda)\right). \; S3$$

By combining equations S3, S2 and S1, we can thus calculate the effective charge-carrier mobility as:

$$\phi\mu = -\epsilon_0 c(n_A + n_B)\frac{A_{eff}hc}{Ee\lambda\left(1 - R_{pump}(\lambda)\right)\left(1 - T_{pump}(\lambda)\right)}\left(\frac{\Delta T(t=0)}{T}\right). \quad S4$$

Given that the branching ratio $0 \leq \phi \leq 1$, the calculated effective charge-carrier mobility is always an underestimate; only in the case of full conversion of photons to free charges does our value reflect the true mobility. In addition, our calculated value of μ arises from changes in photoconductivity due to both electrons and holes, meaning we calculate an overall sum mobility.

Exploratory Synthesis – $CuAgBiI_5$ (and Cu_2AgBiI_6) discovery

For the exploratory synthesis of the CuI–AgI–BiI_3 phase space CuI (99.999% Sigma–Aldrich), AgI (99.999% Alfa Aesar) and BiI_3 (synthesised as previously reported)[1] powders were ground together in a pestle and mortar and pressed in to a pellet with a 5 mm diameter. The pellet was sealed in an evacuated fused silica tube. The fused silica tubes had a 6 mm internal diameter, 1 mm thick walls and were sealed at 10^{-4} mbar to 15 cm in length. The sample in the tube was cooled in liquid N_2 during the evacuation and sealing process to avoid sublimation of the iodides. The sealed tube was placed upright inside a furnace with enclosed heating elements and heated to 610°C for 16 hours to melt and mix the powders. The furnace was cooled to 350°C at 5°C/min and kept there for 5 days. The furnace was then cooled to room temperature at 5°C/min. The tube was scored and carefully broken open to retrieve the pellet, which was then ground, resulting in a dark red powder. To identify the compounds synthesised we carry out phase identification using Pawley fits to powder X-ray diffraction (PXRD) data to extract lattice parameters of phases with LaB_6 as an internal standard and compare them to those reported in the literature. We also perform SEM and TEM EDX as a direct measurement of elemental composition using CuI, AgI and BiI_3 powders as standards.

We searched for quaternary compounds in four locations in the CuI–AgI–BiI_3 phase field (Figure S1); compositions $Cu(Ag_{1.5}Bi_{0.5})I_4$ and $Cu_{1.5}Ag_{0.5}BiI_5$, as well as the solid solution lines $Cu_xAg_{1-x}BiI_4$ (between $Cu_{1/4}Bi_{1/4}I$ and $Ag_{1/4}Bi_{1/4}I$) and $Cu_{4x}(AgBi)_{1-x}I_4$ (between CuI and $Ag_{1/4}Bi_{1/4}I$).We note that different conditions, such as temperature, may lead to the isolation of phase pure materials with different compositions.

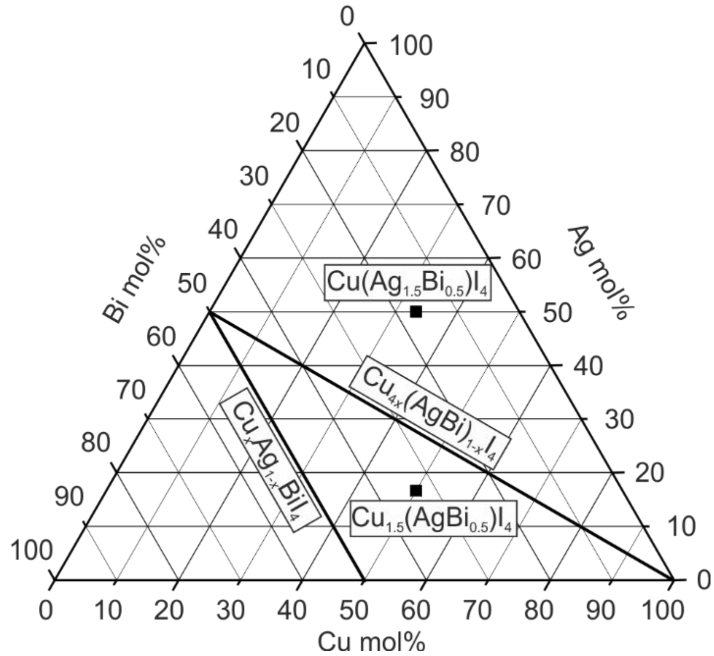

Figure S1. The areas of the CuI–AgI–BiI_3 phase space explored with solid state synthesis during the investigation.

$Cu(Ag_{1.5}Bi_{0.5})I_4$ is the composition where the material would have the same tetrahedral and octahedral occupancies as spinel $MgAl_2O_4$ whilst satisfying charge balance. The PXRD pattern from the $Cu(Ag_{1.5}Bi_{0.5})I_4$ reaction showed that no quaternary Cu–Ag–Bi–I phases formed under the synthesis conditions used. We identified two phases, and their lattice parameters were refined using a Pawley fit (Figure S2). One phase was identified as a $Cu_{1-y}Ag_yI$ zinc blende phase crystallizing in the cubic $F\bar{4}3m$

space group with a lattice parameter of a = 6.2868(5) Å. Using the data from Chessin *et al.*[10] this lattice parameter corresponds to a mixed Cu^+ and Ag^+ zinc blende phase of $Cu_{0.45}Ag_{0.55}I$ (y = 0.45). The other phase was fitted to a small trigonal unit cell with lattice parameters a = 4.3552(4) Å and c = 20.761(2) Å in space group $R\bar{3}m$. The refined lattice parameters are within the range reported for rhombohedral Ag-rich $x < 0$ $Ag_{1-3x}Bi_{1+x}I_4$ phases.[11-13]

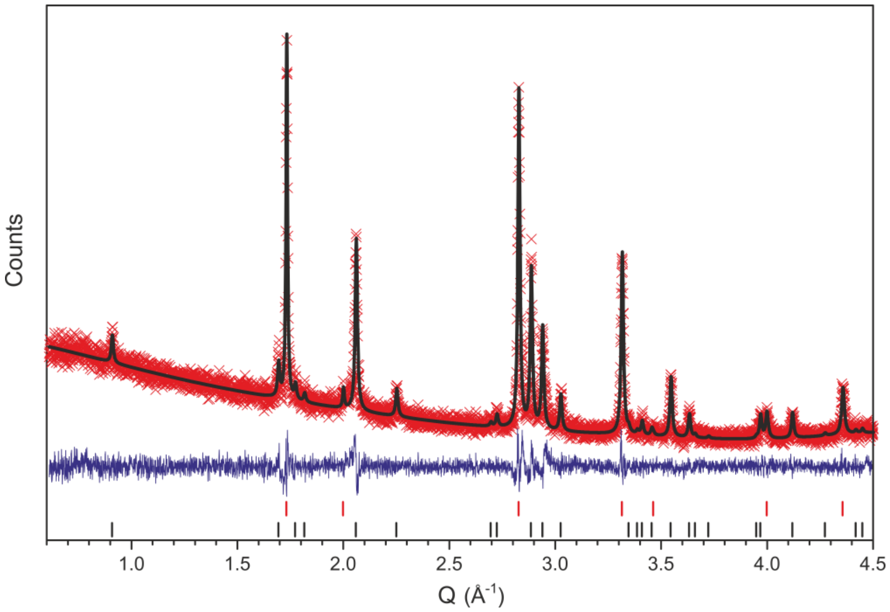

Figure S2. A Pawley fit to laboratory PXRD data of the synthesis with nominal composition $Cu(Ag_{1.5}Bi_{0.5})I_4$. The red tick marks correspond to the $Cu_{0.45}Ag_{0.55}I$ impurity peaks, and the black tick marks to the rhombohedral Ag-rich $x < 0$ $Ag_{1-3x}Bi_{1+x}I_4$ phases.

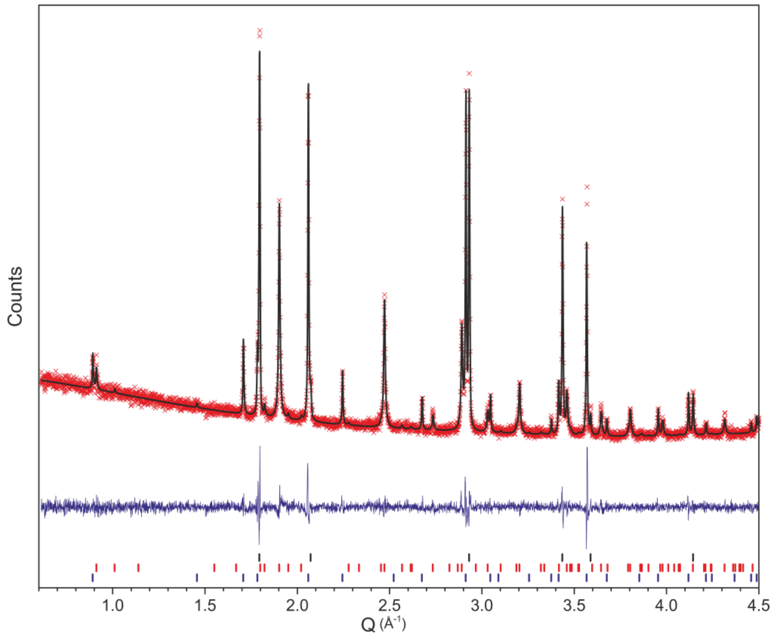

Figure S3. A Pawley fit to the laboratory PXRD pattern of nominal composition $Cu_{1.5}Ag_{0.5}BiI_5$. The tick marks for the CuI impurity, BiI_3 impurity, and the cubic $Fd\bar{3}m$ phase are black, red and blue, respectively. The lattice parameter of the $Fd\bar{3}m$ phase (a = 12.2050(3) Å) is larger than that reported for $CuBiI_4$ (a = 12.1580(2) Å) and smaller than that reported for $AgBiI_4$ (a = 12.21446(4) Å), suggesting a quaternary phase containing both Cu and Ag.

$Cu_{1.5}Ag_{0.5}BiI_5$ is a composition which gives a 1:1 tetrahedral-to-octahedral cation ratio. A Pawley fit to the PXRD pattern from the $Cu_{1.5}Ag_{0.5}BiI_5$ reaction shows the presence of CuI and BiI_3 impurities and a $Fd\bar{3}m$ cubic phase (Figure S3). The lattice parameter of the $Fd\bar{3}m$ phase (a = 12.2050(3) Å) is larger than that reported for $CuBiI_4$ (a = 12.1580(2) Å) and smaller than that reported for $AgBiI_4$ (a = 12.21446(4) Å), suggesting a quaternary phase containing both Cu^+ and Ag^+. However, the large amounts of impurity phases show that the nominal composition used is far from the new targeted quaternary phase.

The $Cu_xAg_{1-x}BiI_4$ line corresponds to compositions between the known materials $CuBiI_4$ and $AgBiI_4$. The PXRD patterns of samples synthesized at x values (x = 0.25, 0.33, 0.50, 0.66, 0.75) can all be fitted to a mixture of a $F\bar{4}3m$ $Cu_{1-y}Ag_yI$ zinc blende, BiI_3 and a cubic $Fd\bar{3}m$ phase (Figure S4). The lattice parameters of the $Cu_{1-y}Ag_yI$ zinc blende and the cubic $Fd\bar{3}m$ phase across the series were refined with respect to a LaB_6 internal standard (Figure S5). The lattice parameter of the cubic $Fd\bar{3}m$ phase is refined as a = 12.2034(3) Å at nominal x = 0.25 ($Cu_{0.25}Ag_{0.75}BiI_4$) suggesting a quaternary phase containing both Cu^+ and Ag^+. The lattice parameter grows as x increases which indicates an increase in Ag^+ content until it levels off with a lattice parameter of a = 12.2143(5) Å for nominal compositions $x \geq 0.66$ ($Cu_{0.66}Ag_{0.33}BiI_4$), consistent with that reported for $AgBiI_4$ (a = 12.21446(4) Å). The lattice parameter of the new quaternary phase identified for nominal composition $Cu_{1.5}Ag_{0.5}BiI_5$ lies within this range of lattice parameters. The lattice parameter of the $Cu_{1-y}Ag_yI$ zinc blende phase also shows a small increase over the series suggesting a small amount of Ag^+ is being substituted into the structure for Cu^+ (< 5 mol% at x = 0.75). The range of lattice parameters over the series suggests that a range of quaternary Cu–Ag–Bi–I phases may exist under these synthetic conditions, however, no pure quaternary phases were isolated on the $Cu_xAg_{1-x}BiI_4$ line.

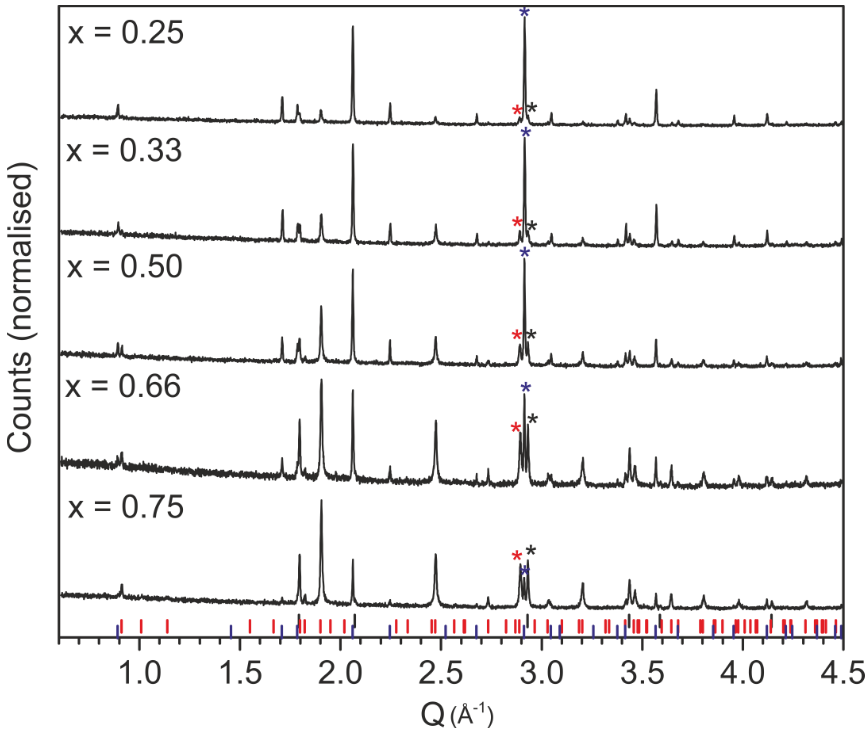

Figure S4. The laboratory PXRD pattern of the $Cu_xAg_{1-x}BiI_4$ series. These PXRD patterns are indexed to a mixture of three phases; a zinc blende $Cu_{1-y}Ag_yI$ phase (black tick marks and asterisks), BiI_3 (red tick marks and asterisks), and a cubic $Fd\bar{3}m$ phase (blue tick marks and asterisks). The peaks marked around 2.9 Å^{-1} are used to show the presence of all three phases in all of the patterns.

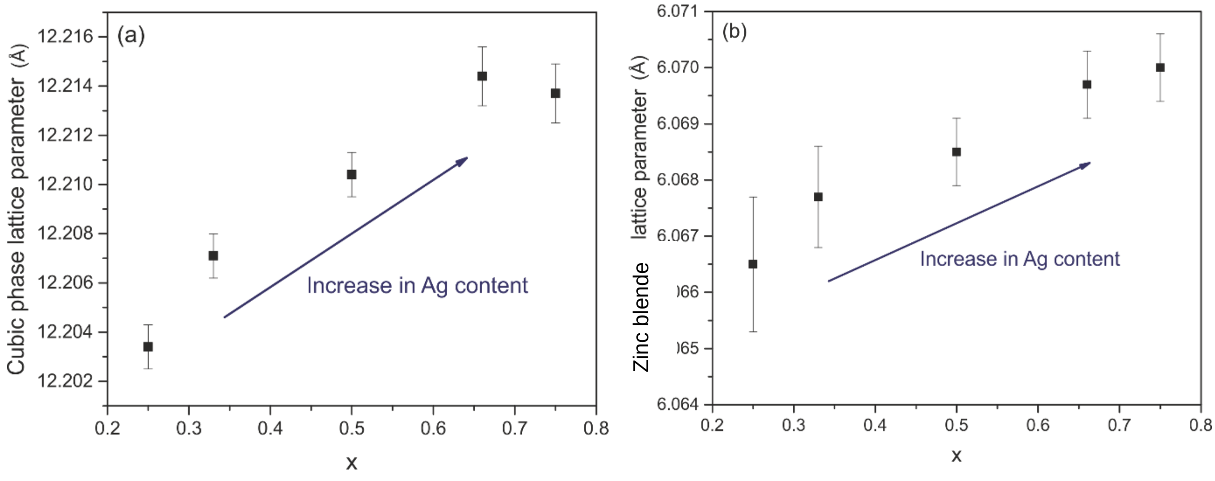

Figure S5. Refined lattice parameters of (a) the $Fd\bar{3}m$ cubic phase and (b) zinc blende $Cu_{1-y}Ag_yI$ phase over the $Cu_xAg_{1-x}BiI_4$ solid solution series. Lattice parameters were refined against an internal LaB_6 standard using a Pawley fit.

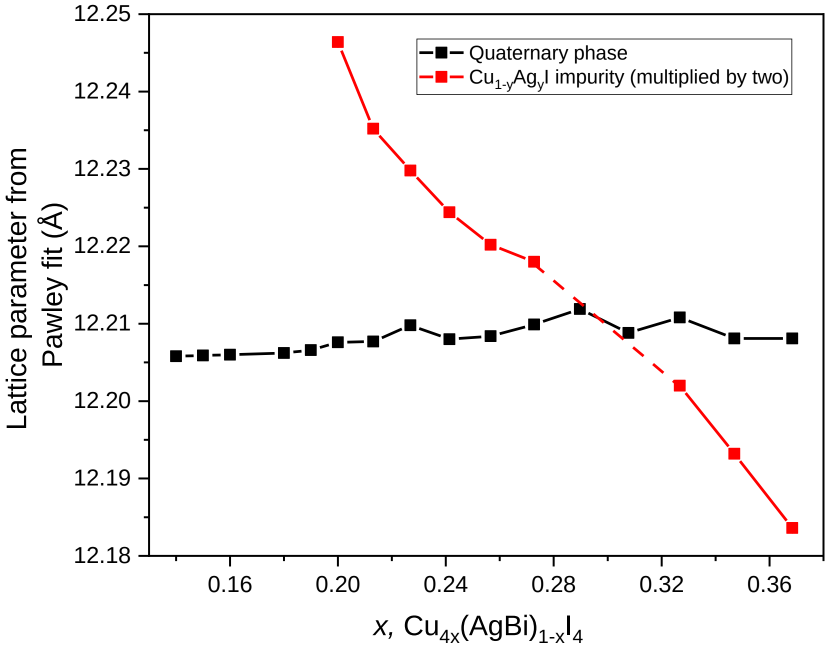

Figure S6. The $Cu_{4x}(AgBi)_{1-x}I_4$ solid solution corresponds to a line of compositions between the known phases CuI and $AgBiI_4$. Samples were synthesized at x = 0.14, 0.15, 0.16, 0.18, 0.2, 0.21, 0.23, 0.24, 0.26, 0.27, 0.29, 0.31, 0.33, 0.35, 0.37, 0.43, 0.5. The refined lattice parameters of the main cubic $Fd\bar{3}m$ phase and $Cu_{1-y}Ag_yI$ impurity (multiplied by 2) are shown in this figure. For $0.16 < x < 0.18$ there could be a pure quaternary phase. For $0.29 \leq x \leq 0.31$ $Cu_{4x}(AgBi)_{1-x}I_4$ the $Cu_{1-y}Ag_yI$ impurity lattice parameters are half that of the quaternary phase peaks, showing that these samples could be pure, but alternatively it could be that the $Cu_{1-y}Ag_yI$ impurity peaks cannot be resolved from the main phase peaks.

The $Cu_{4x}(AgBi)_{1-x}I_4$ solid solution corresponds to a line of compositions between the known phases CuI and $AgBiI_4$. Samples were synthesized at x = 0.14, 0.15, 0.16, 0.18, 0.2, 0.21, 0.23, 0.24, 0.26, 0.27, 0.29, 0.31, 0.33, 0.35, 0.37, 0.43, 0.5. For $0.14 \le x \le 0.16$ samples the PXRD data is fitted to a mixture of a cubic unit cell with space group $Fd\bar{3}m$ and a small trigonal unit cell with space group $R\bar{3}m$. The cubic $Fd\bar{3}m$ phase has a constant lattice parameter for $0.14 \le x \le 0.16$, with an average value of a = 12.206(2) Å, suggesting a quaternary phase containing both Cu^+ and Ag^+ (Figure S6). The trigonal $R\bar{3}m$ phase also has constant lattice parameters with average values of a = 4.3530(4) Å, c = 20.764(2) Å, consistent with the reported $x < 0$ $Ag_{1-3x}Bi_{1+x}I_4$ phases (Figure S7a). For the $0.18 \le x \le 0.37$ samples there remains a new quaternary cubic $Fd\bar{3}m$ phase with lattice parameter a = 12.209(1) Å. There are no PXRD peaks associated with the $x < 0$ $Ag_{1-3x}Bi_{1+x}I_4$ phases however there is a $Cu_{1-y}Ag_yI$ zinc blende impurity which increases in weight percentage for higher values of x (Figure S7b). This suggested that a pure quaternary phase could be isolated near to $0.16 < x < 0.18$.

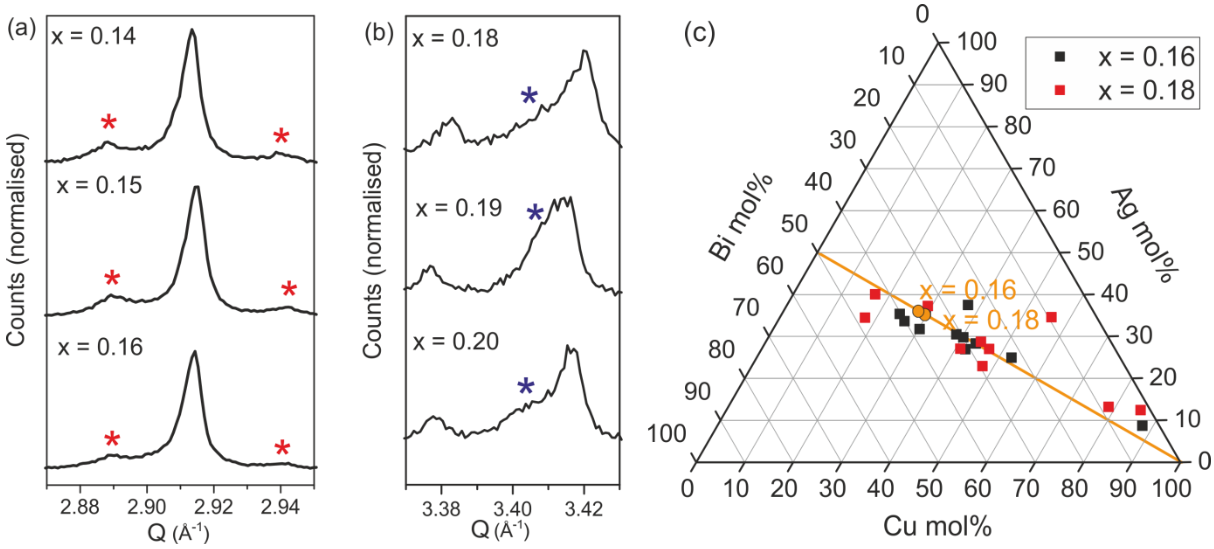

Figure S7. (a) A selected Q range of the PXRD pattern for the $0.14 \le x \le 0.16$ $Cu_{4x}(AgBi)_{1-x}I_4$ samples. The red asterisks highlight peaks due to a rhombohedral $R\bar{3}m$ Ag-rich $Ag_{1-3x}Bi_{1+x}I_4$ impurity phase, no $Cu_{1-y}Ag_yI$ impurity is seen. (b) A selected Q range of the PXRD pattern for the $0.18 \le x \le 0.20$ samples. The blue asterisks highlight peaks due to a $Cu_{1-y}Ag_yI$ impurity phase, no rhombohedral $R\bar{3}m$ Ag-rich $Ag_{1-3x}Bi_{1+x}I_4$ impurity phase is seen. (c) TEM EDX showing the measured composition of particles from the x = 0.16 and 0.18 samples, showing them to be compositionally inhomogeneous along the solid solution line.

Additionally, an interesting point in the series lies between $0.29 \le x \le 0.31$ in which the PXRD appears to only show a new quaternary cubic $Fd\bar{3}m$ phase and the inclusion of the $Cu_{1-y}Ag_yI$ zinc blende impurity only slightly improves the Pawley fit by improving peak shapes (Figure S8a). The lattice parameter of the $Cu_{1-y}Ag_yI$ zinc blende impurity phase changes with composition and for $0.29 \le x \le 0.31$ the impurity peaks are aligned with the main phase peaks and cannot be deconvoluted from the PXRD pattern during the Pawley fit. For this region, the $Cu_{1-y}Ag_yI$ zinc blende lattice parameter is interpolated to be half the lattice parameter of the main cubic $Fd\bar{3}m$ phase (Figure S6). As we could not conclusively identify the presence of the $Cu_{1-y}Ag_yI$ zinc blende phase, it was considered possible that this is a region where a pure quaternary phase also exists. TEM EDX was carried out on particles from samples x = 0.16, 0.18 (Figure S7c) and x = 0.27, 0.29, 0.31, 0.33 (Figure S8b) to find the composition of the targeted quaternary cubic $Fd\bar{3}m$ phases. It was found that an individual sample

showed large compositional inhomogeneity consisting of particles with compositions spread along the $Cu_{4x}(AgBi)_{1-x}I_4$ solid solution. The observation that a single phase can fit the XRD pattern despite the compositional inhomogeneity seen in the EDX indicates that the response of the unit cell size to this inhomogeneity is limited.

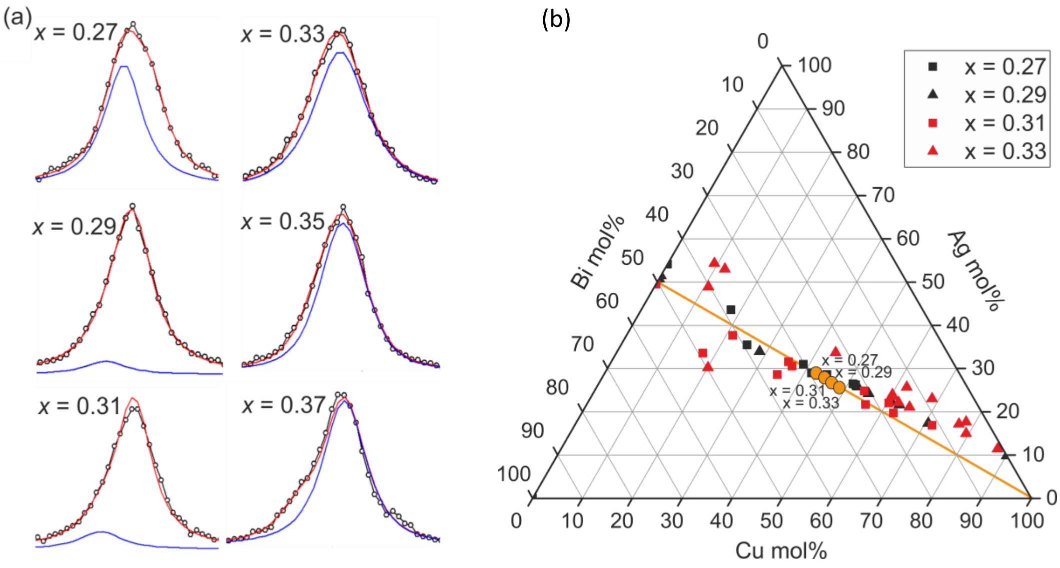

Figure S8. The $Cu_{4x}(AgBi)_{1-x}I_4$ solid solution corresponds to a line of compositions between the known phases CuI and $AgBiI_4$. Samples were synthesized at x = 0.14, 0.15, 0.16, 0.18, 0.2, 0.21, 0.23, 0.24, 0.26, 0.27, 0.29, 0.31, 0.33, 0.35, 0.37, 0.43, 0.5. (a) The Pawley fit of the peak which would include the largest CuI impurity peak for $0.27 \le x \le 0.37$. The black line is the measured intensity, the red line is the calculated PXRD pattern, the blue line is the contribution of the CuI impurity phase. For $x > 0.27$ and x = 0.31 the inclusion of CuI only slightly improves peak shape; which makes the samples be interpreted as pure. (b) The TEM EDX composition of particles from the $0.27 \le x \le 0.33$ samples, showing them to be compositionally inhomogeneous along the $Cu_{4x}(AgBi)_{1-x}I_4$ solid solution line.

Following the exploratory synthesis, we continued to optimise synthetic parameters around the regions identified as pure by PXRD (x = 0.2 $CuAgBiI_5$ and x = 0.33 Cu_2AgBiI_6). We have previously reported the optimised procedure to synthesise Cu_2AgBiI_6 powder without going in to detail of the exploratory synthesis.[14] We showed there that it was found to be crucial to quench the Cu_2AgBiI_6 powder from 350°C, and avoiding temperatures which would melt the compound, to avoid the compositional inhomogeneity, and the same is true here for $CuAgBiI_5$, which leads to our optimised $CuAgBiI_5$ synthesis.

Fitting $CuBiI_4$ PXRD data to structures with the spinel and $CdCl_2$ octahedral motifs

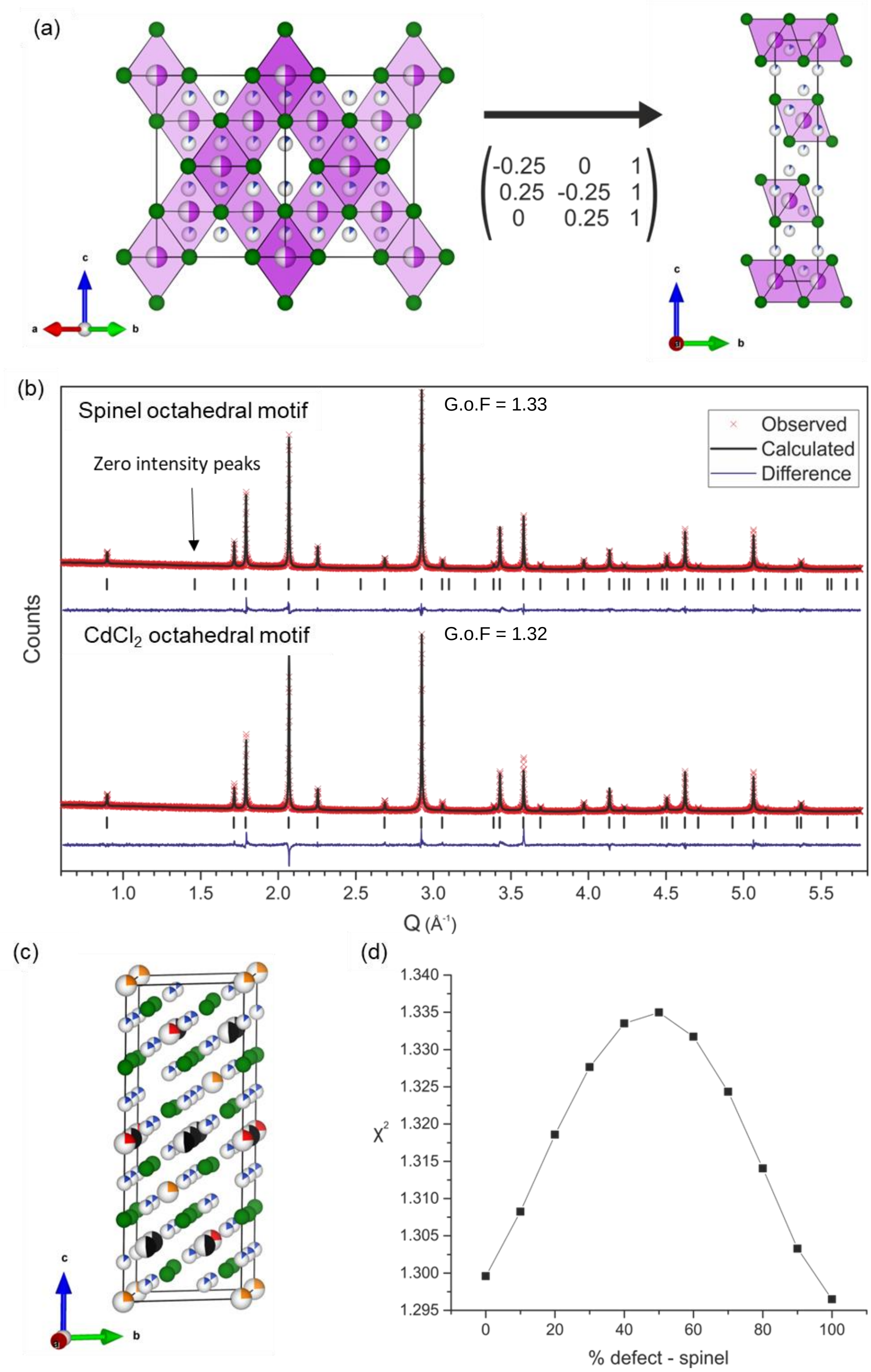

Figure S9. (a) The $CuBiI_4$ structure as reported by Fourcroy *et al.*[15] transformed in to a structure with the $CdCl_2$ octaledral motif, using the transformation matrix shown. (b) The Rietveld refinements of laboratory PXRD data collected at room temperature for the $CuBiI_4$ material, fitted to the reported cubic $Fd\overline{3}m$ $CuBiI_4$ structure with a spinel octahedral motif (top) and the new $CuBiI_4$ structure with the

$CdCl_2$ octahedral motif (bottom). (c) The $CuBiI_4$ structural model built to represent a 50-50 mixture of materials with the spinel and $CdCl_2$ octahedral motifs. This is achieved by fixing the octahedral occupancy of octahedral sites common to both octahedral motifs. Then the octahedral sites that are only present in the spinel and $CdCl_2$ octahedral motifs are fixed to half this value. Octahedral sites shared by both types of octahedral motifs are in black, the spinel in orange, and the $CdCl_2$ in red. The Cu sites (blue) fill every tetrahedral hole facilitated by the iodide sub-lattice (green). For a more in-depth description please see our work on $AgBiI_4$.[1] (d) The change in goodness of fit parameter for Rietveld refinements with respect to the ratio of defect-spinel:$CdCl_2$ cation motif in the model used in the refinement. The structural parameters of this new $CuBiI_4$ structure are shown in Table S1.

Table S1. Crystallographic data for the refinement of $CuBiI_4$ with the $CdCl_2$ octahedral motif.

Crystallographic Data ($CuBiI_4$ Powder)	
Source	laboratory X-ray
Formula weight (g/mol)	1166.4
Temperature (K)	293
Wavelength (Å)	1.7890
Crystal system	Trigonal
Space group (No.)	166
a, b, c, α, β, γ	4.2990(2) Å, 4.2990(2) Å, 21.0597(11) Å, 90°, 90°, 120°
V (Å^3)	337.07(3)
Z	1
d-space range (Å)	1.09201-10.27186
χ^2	1.75
R_p	7.65
R_{wp}	9.82

$R_p = \frac{\sum|Y_{o,m}-Y_{c,m}|}{\sum Y_{o,m}}$, $R_{wp} = \sqrt{\frac{\sum w_m[Y_{o,m}-Y_{c,m}]^2}{\sum w_m Y_{o,m}^2}}$, $\chi^2 = \frac{\sum w_m[Y_{o,m}-Y_{c,m}]^2}{M-N}$, where $Y_{o,m}$ and $Y_{c,m}$ are the observed and calculated data respectively at data point m; M the number of data points, N the number of parameters, w_m the weighting given to data point m; for counting statistics $w_m = \frac{1}{\sigma(Y_{o,m})^2}$, where $(Y_{o,m})$ is the error in $Y_{o,m}$

Table S2. The structural information of $CuBiI_4$ with the $CdCl_2$ octahedral motif, refined against laboratory PXRD data.

Site	Atom	x	y	z	Occupancy	U (Å^{2}x10^3)	Wyckoff Position	Point group (Hermann-Mauguin)
I1	I	0	0	0.24622(12)	1	34(2)	6c	3m
Bi1	Bi	0	0	0	0.5	51(2)	3a	$\bar{3}$m
Cu1	Cu	0	0	3/8	0.15	42(4)	6c	3m
Cu2	Cu	0	0	1/8	0.09	42(4)	6c	3m

Table S3. Bond distances and angles for the $CuBiI_4$ structure with the $CdCl_2$ octahedral motif.

Bond distances (Å)			Bond angles (°)			
Bi1	I1	3.087(2)	I1	Bi1	I1	88.28(4)
I1	Cu2	2.553(3)	I1	Bi1	I1	91.72(4)
I1	Cu1	2.712(3)	I1	Cu1	I1	111.09(6)
I1	Cu1	2.660(1)	I1	Cu1	I1	107.81(6)
I1	Cu1	2.6071(8)	I1	Cu2	I1	107.82(6)
I1	I1	4.170(5)	I1	Cu2	I1	111.07(5)
I1	I1	4.2990(2)	I1	I1	I1	58.97(4)
I1	I1	4.430(5)	I1	I1	I1	62.06(7)
			I1	I1	I1	60.97(4)
			I1	I1	I1	58.06(7)
			I1	I1	I1	60.000(3)

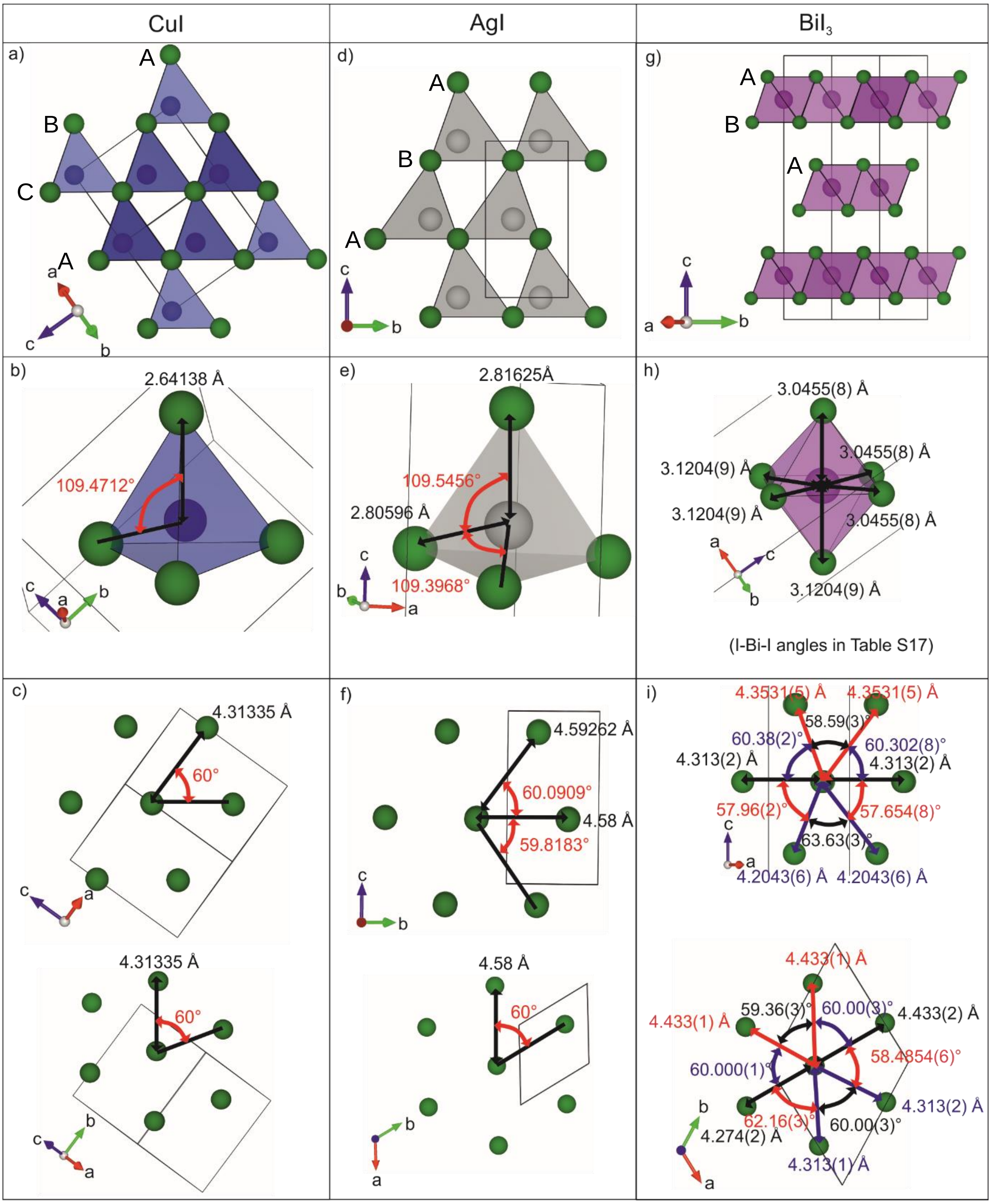

Figure S10. The crystal structures of room temperature binaries zinc blende CuI (a),[16] wurtzite AgI (d),[17] and BiI_3 (g),[18] showing the stacking sequence of the iodide sub-lattice. The metal coordination environments are shown for; (b) tetrahedral Cu^+ in CuI, (e) tetrahedral Ag^+ in AgI, and (h) octahedral Bi^{3+} in BiI_3. The I–I distances and I–I–I angles of the iodide sub-lattices of CuI, AgI and BiI_3 are shown in (c), (f), and (i) respectively. Green, blue, grey, and pink spheres represent the I^-, Cu^+, Ag^+ and Bi^{3+} ions, respectively.

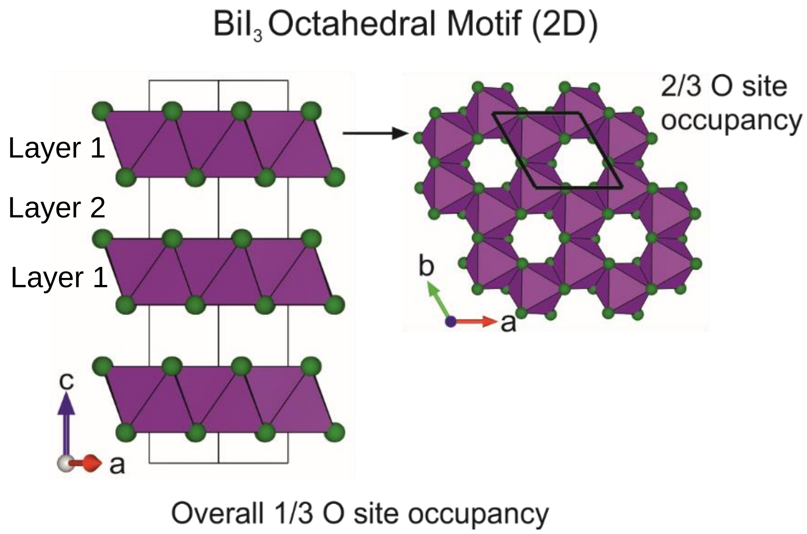

Figure S11. The crystal structure of BiI_3,[18] showing the arrangement of the occupied octahedral interstitial in filled layers. In layer 1, which accounts for half of the octahedral interstitials, two thirds of the interstitials are occupied by Bi^{3+} with an atomic occupancy of 1. In layer 2, which accounts for the other half of the octahedral interstitials, there is no octahedral occupancy. Therefore, the overall octahedral interstitial occupancy is (1/2 x 2/3 x 1) + (1/2 x 0) = 1/3, as is evident from the composition BiI_3.

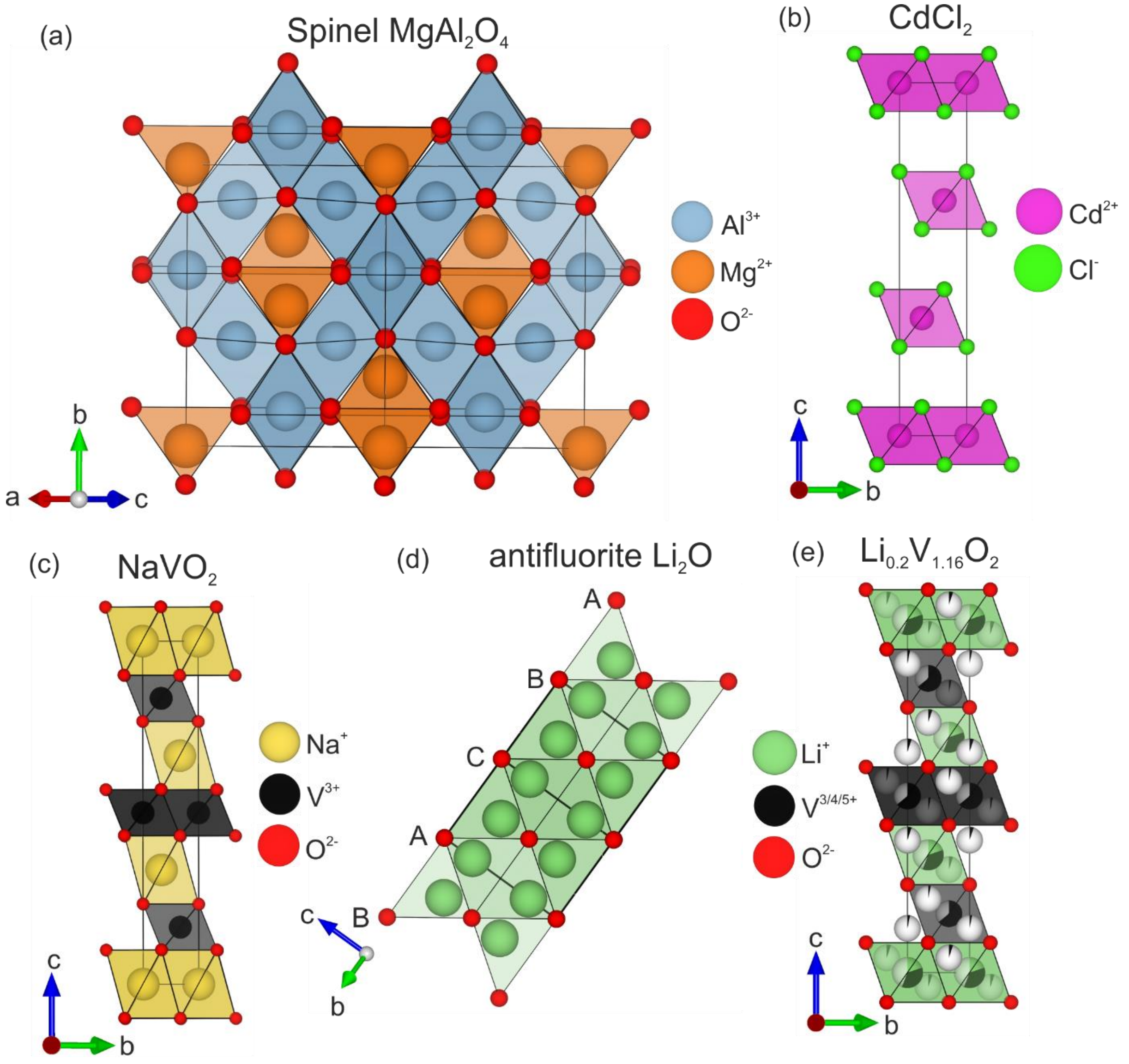

Figure S12. The structures of spinel, $CdCl_2$, $NaVO_2$, antifluorite Li_2O and $Li_{0.2}V_{1.16}O_2$.[19-23]

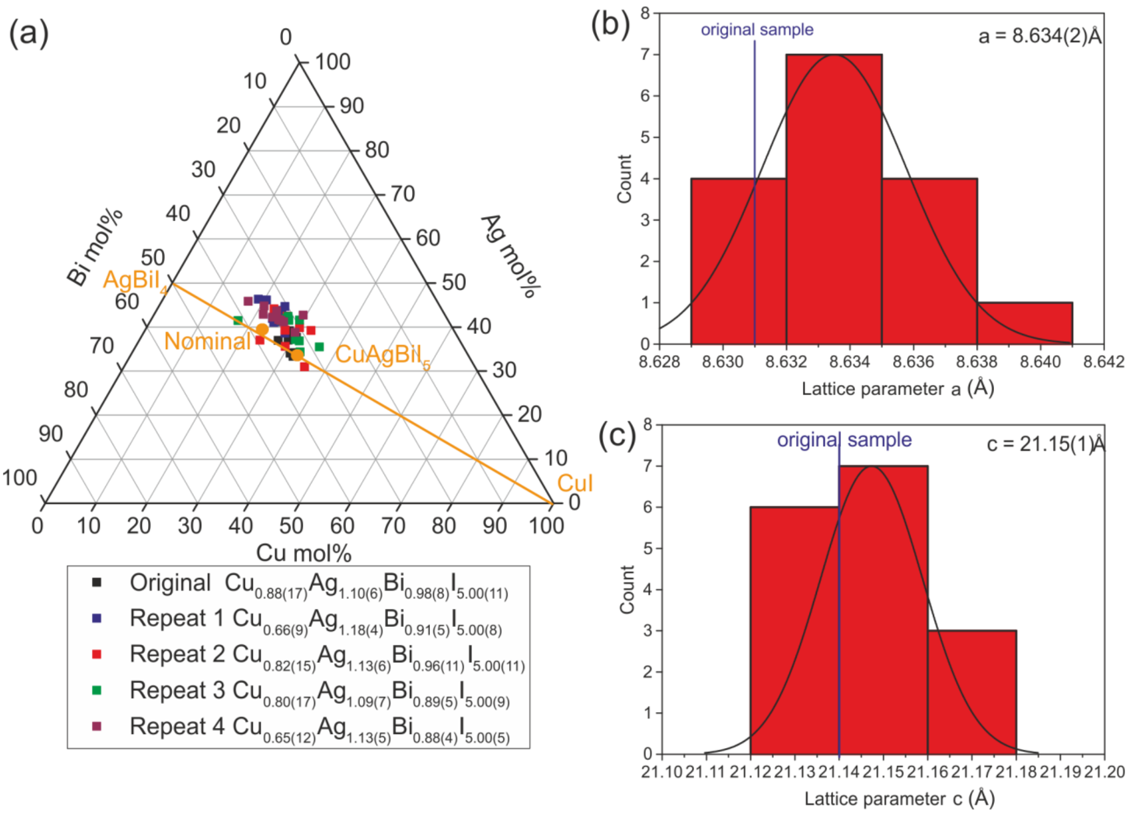

Figure S13. (a) TEM EDX compositions of particles taken from the original $CuAgBiI_5$ sample and repeated syntheses. The *a* and *c* lattice parameters obtained by fitting a $R\bar{3}m$ large trigonal unit cell in a Pawley fit are shown in b and c, respectively. These samples consisted of 0.25g batches which were combined in to a larger batch for NPD.

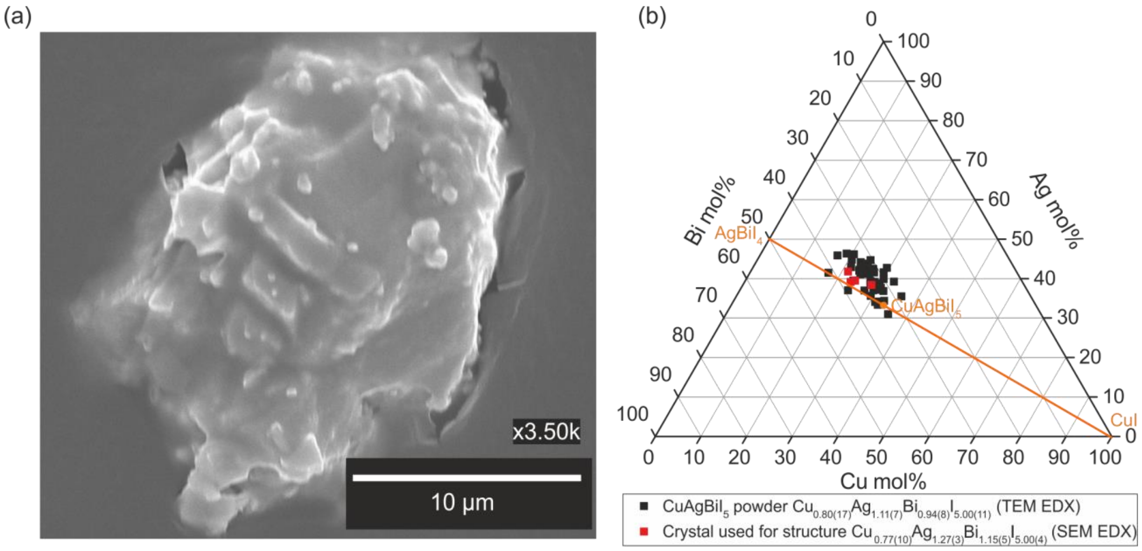

Figure S14. (a) SEM image of the $CuAgBiI_5$ crystal used for the SCXRD structure. the crystal was picked out of the $CuAgBiI_5$ powder sample. (b) The SEM EDX measurements of the crystal (red points) compared to the TEM EDX measurements of powder samples (black).

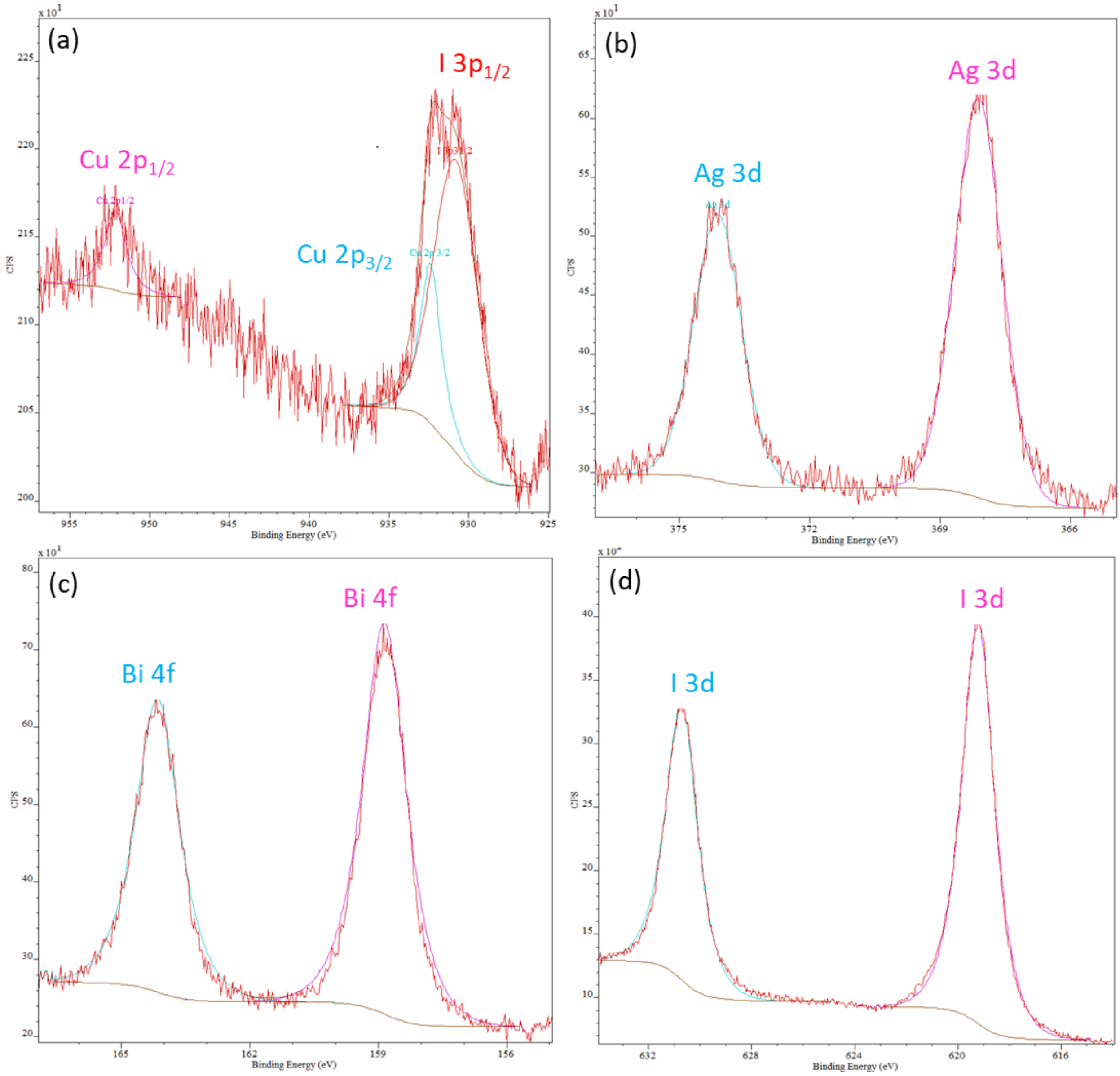

Figure S15. The fitting of (a) Cu 2p, (b) Ag 3d, (c) Bi 4f, and (d) I 3d core levels of X-ray photoelectron spectroscopy (XPS) data collected for $CuAgBiI_5$ bulk sample. The fitting of Cu $2p_{3/2}$ is complicated by the presence of the I $3p_{1/2}$, however we fit the Cu $2p_{1/2}$ first, and use the known spin orbit split energy. The identification of the charge state of the Cu as Cu^+ is supported by the lack of any satellite feature of the Cu 2p.

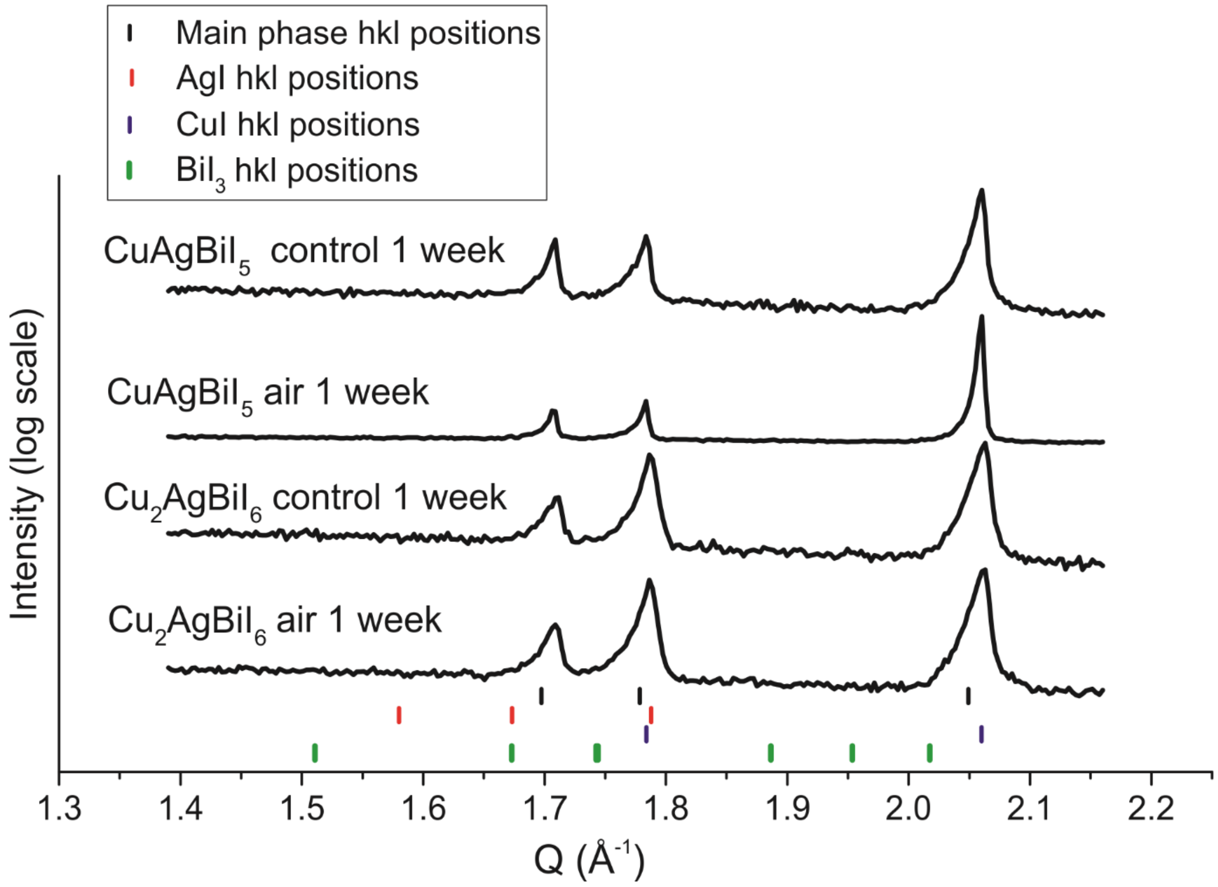

Figure S16. PXRD patterns measured on capillaries containing $CuAgBiI_5$ (a) and Cu_2AgBiI_6 (b) powders sealed under air and exposed to the solar spectrum for 1 week. showing no signs of decomposition compared to the controls kept in the dark and air for 1 week.

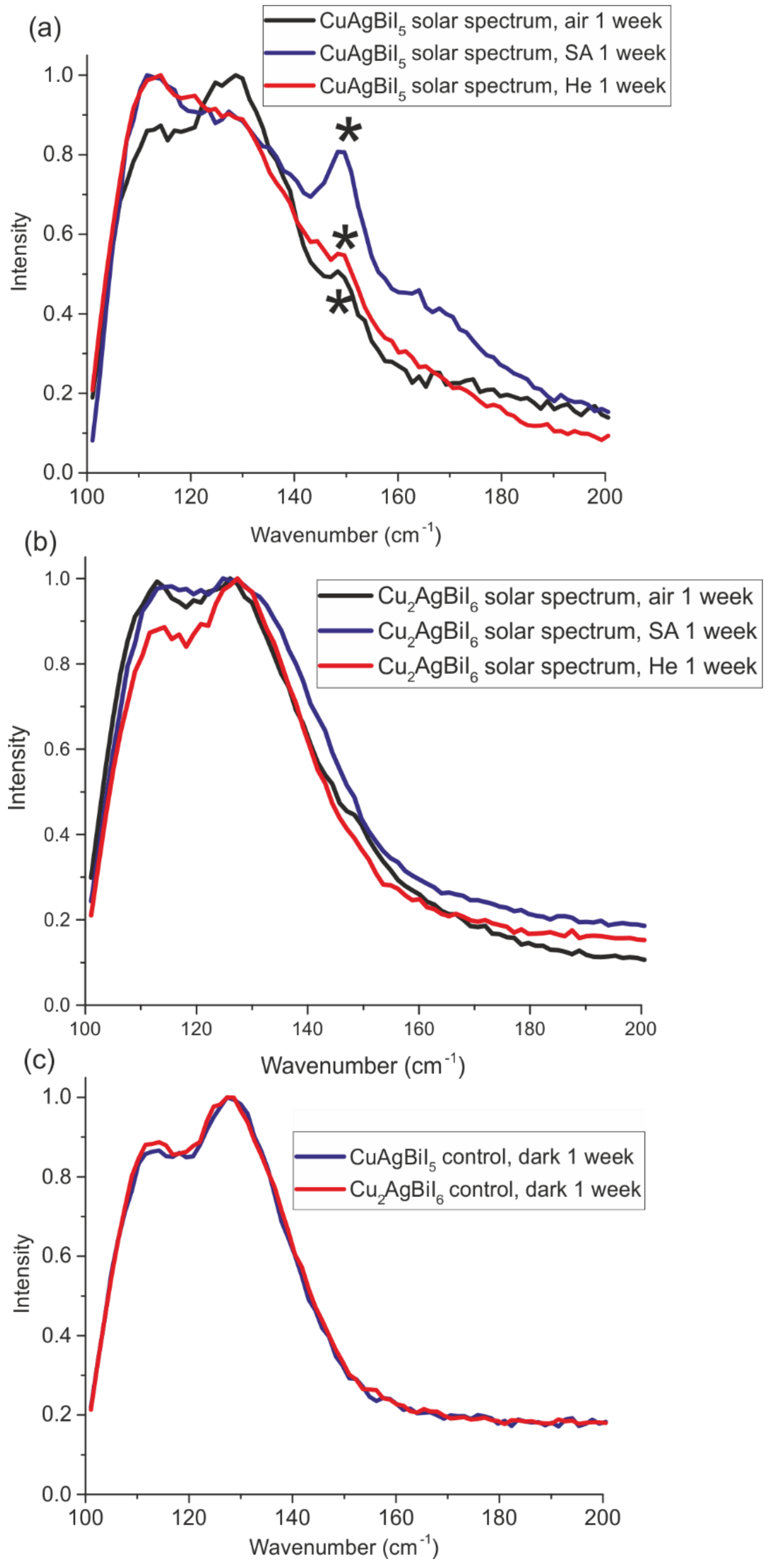

Figure S17. Raman spectroscopy performed on capillaries containing $CuAgBiI_5$ (a) and Cu_2AgBiI_6 (b) powders sealed under air, synthetic air and He and exposed to the solar spectrum for 1 week. The asterisks show the presence of a peak for $CuAgBiI_5$ which was not observed in the control sample (c). Cu_2AgBiI_6 showed no signs of decomposition.

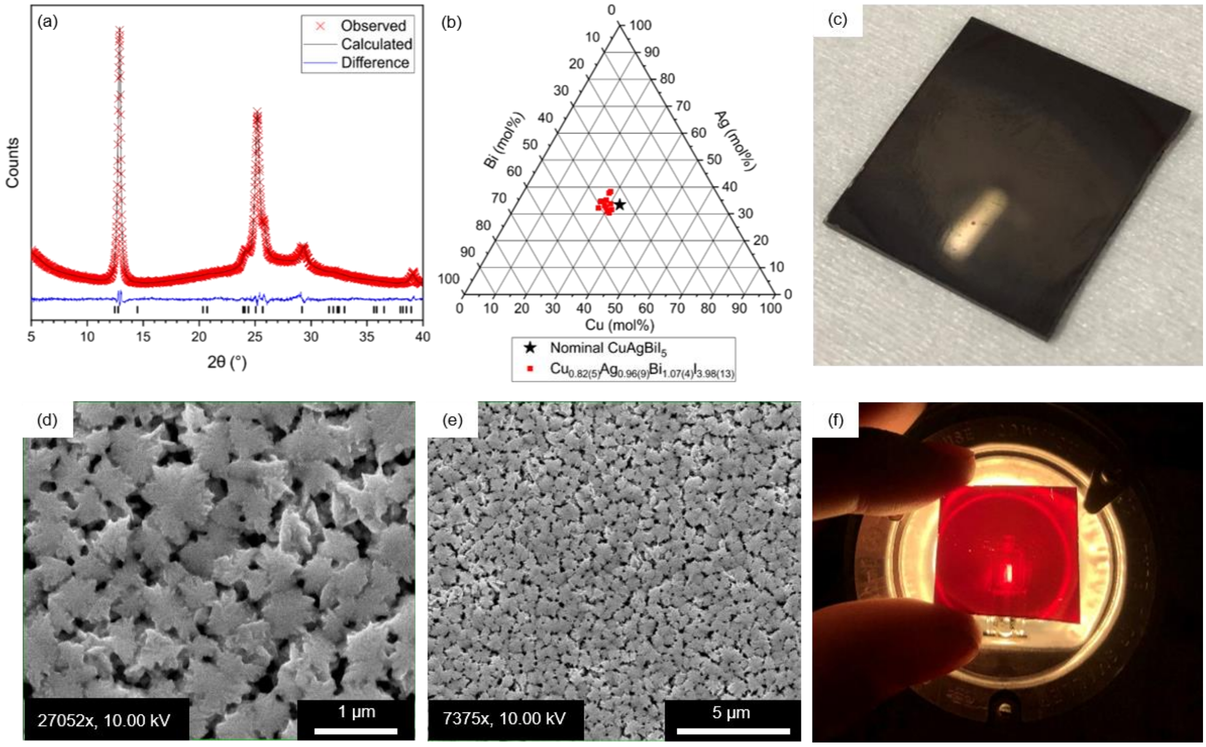

Figure S18. (a) The Pawley fit of XRD data collected on a $CuAgBiI_5$ thin film deposited on a microscope slide, fitted to a large trigonal cell associated with $CuAgBiI_5$ with refined lattice parameters a = 8.724(1) Å and c = 20.800(5) Å. (b) The composition of 9 points of the film measured by SEM EDX, with an average composition of $Cu_{0.82(5)}Ag_{0.96(9)}Bi_{1.07(4)}I_{3.98(13)}$ corresponding to an iodine deficit of 20(3)%. (c) Image of a $CuAgBiI_5$ film, showing uniformity but a rough surface caused by the morphology seen in the SEM images in (d) and (e). (f) Image of a backlit film, showing the transmission of red light.

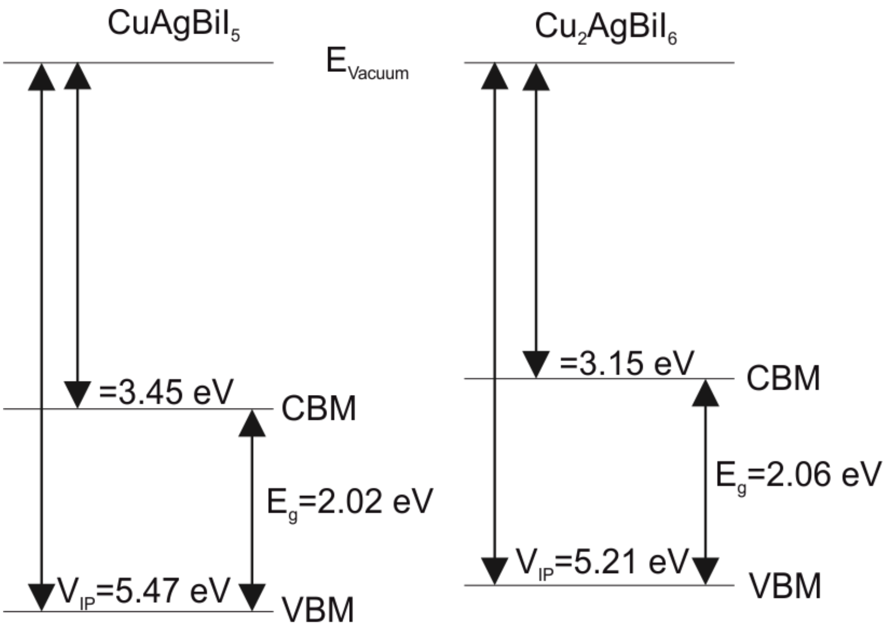

Figure S19. The valence band maximum (VBM) and conduction band minimum (CBM) positions with respect to vacuum measured for $CuAgBiI_5$ and Cu_2AgBiI_6 bulk samples. The ionisation potential are measured on bulk samples and the optical band gap measured on thin films.

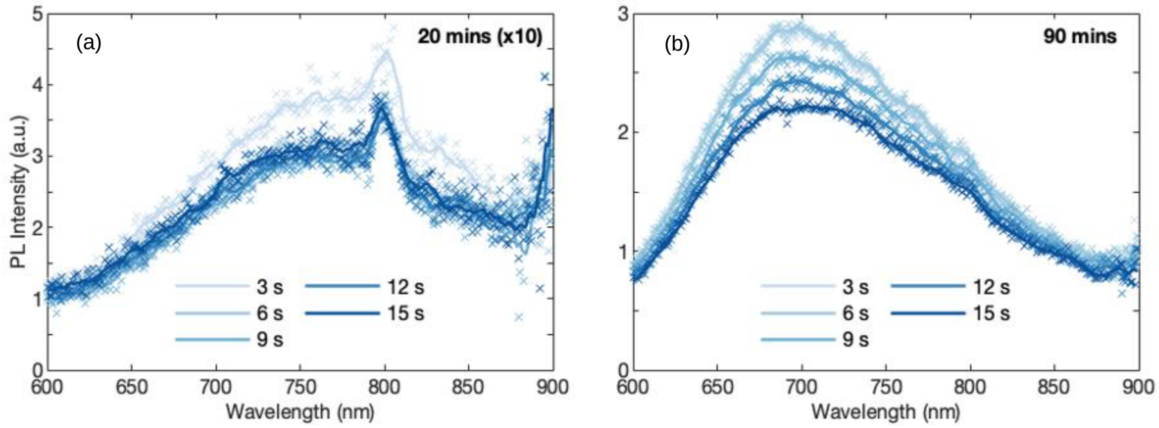

Figure S20. Five steady-state photoluminescence spectra for a thin film of $CuAgBiI_5$ measured under continuous illumination over 15 s by a 398 nm laser at an intensity of 39.0 Wcm^{-2} after being left in air for 20 (a) and 90 (b) minutes, respectively. In both cases the spectra do not change shape but do decrease in intensity over 15 s of continuous illumination.

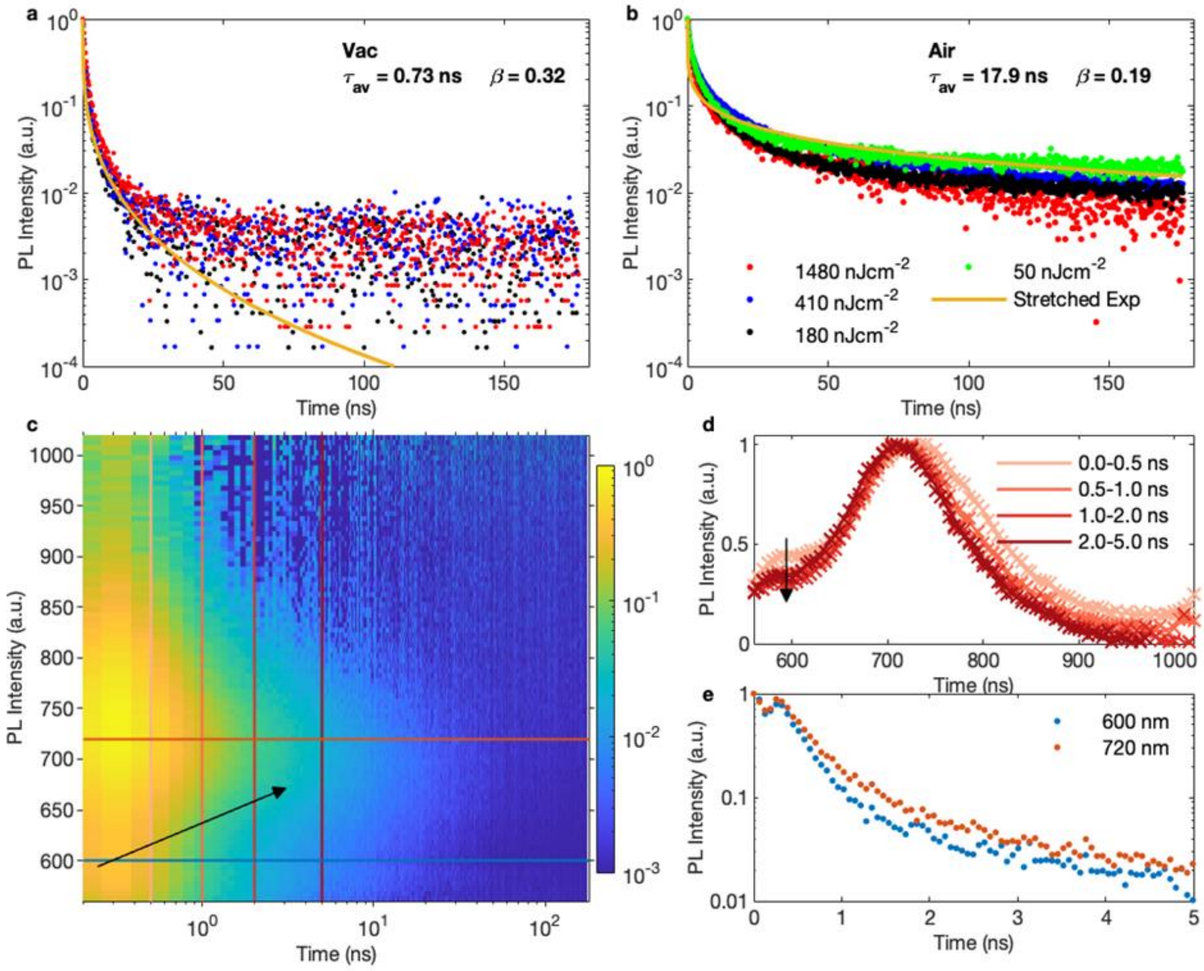

Figure S21. (a) Time-resolved photoluminescence decays for a fresh thin film of $CuAgBiI_5$ measured in vacuum using Time-Correlated Single Photon Counting (TCSPC) following excitation by a 398 nm pulsed laser at a repetition rate of 5 MHz. The lowest-fluence decay was fitted with a stretched exponential, shown in yellow, yielding an average lifetime of 0.73 ns. (b) Time-resolved photoluminescence decays for the same sample as in (a), measured in air after the time-resolved emission spectra shown in (d) – (f). The lowest-fluence decay was again fitted with a stretched exponential, giving an average lifetime of 17.9 ns. (c) Time-resolved emission spectra measured in vacuum using TCSPC. The vertical and horizontal lines indicate spectral and transient decay slices, respectively, which are shown in (d) and (e). The measured values in (d) are shown as crosses, with the lines showing a five-point moving average.

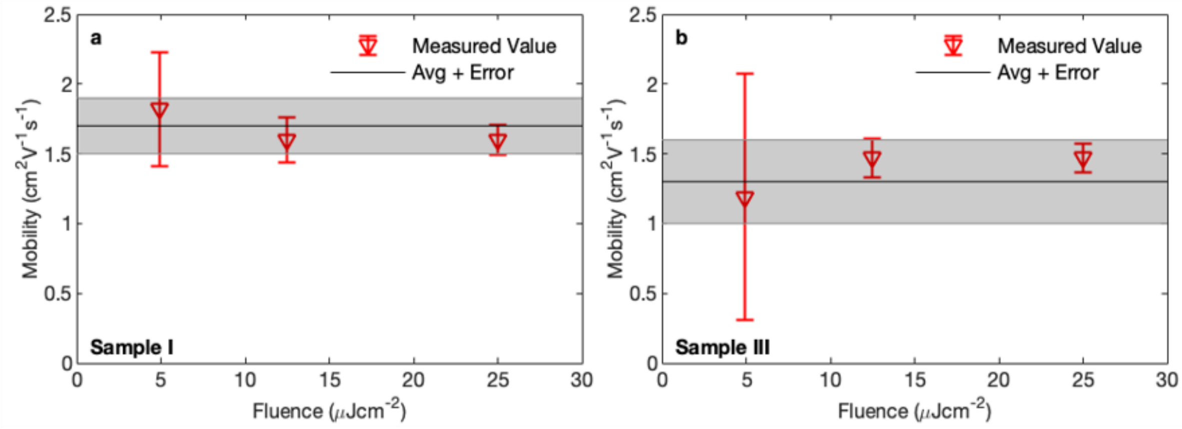

Figure S22. (a,b) Charge-carrier mobilities measured across for two thin films of $CuAgBiI_5$ using Optical-Pump Terahertz-Probe spectroscopy at fluences of 4.9, 12.5, 25 μJcm^{-2}. The measured values and their experimental errors are shown in red with error bars, and the mean and standard error are shown as the black line and grey shaded area.

Table S4. Bond distances and bond angles for the $CuAgBiI_5$ structure refined against room temperature PXRD and NPD combined datasets.

**$CuAgBiI_5$**						
**Bond distances (Å)**			**Bond angles (°)**			
Oct1	I1	3.0570(2)	I1	Oct1	I1	90.167
Oct1	I2	3.0570(2)	I1	Oct1	I1	89.833
Oct2	I1	3.0473(2)	I1	Oct1	I2	90.167
Cu1	I1	2.4923(3)	I1	Oct1	I2	89.833
Cu1	I2	2.6908(3)	I2	Oct1	I2	90.167
I1	I1	4.3020(1)	I2	Oct1	I2	89.833
I1	I2	4.3020(1)	I1	Oct2	I1	90.200
I1	I1	4.3170(1)	I1	Oct2	I1	89.800
I1	I2	4.3170(1)	I1	Cu1	I1	112.140(4)
I1	I1	4.3296(1)	I1	Cu1	I1	106.676(2)
I1	I2	4.3296(1)	I1	Cu1	I2	112.140(4)
			I1	Cu1	I2	106.676(2)

Table S5. The Rietveld refinement parameters of the $CuAgBiI_5$ structures with the defect-spinel octahedral motif to a combination of high-resolution synchrotron PXRD (MAC I11, Diamond Light Course, Oxfordshire, UK) and high resolution NPD (banks 1 and 2, HRPD, ISIS neutron and Muon Source, Oxfordshire, UK).

Crystallographic Data ($CuAgBiI_5$ Powder)

Source	synchrotron X-ray, neutron time of flight (TOF)
Formula weight (g/mol)	4834.67
Temperature (K)	293
Wavelength (Å)	0.825898 (synchrotron)
Crystal system	Trigonal
Space group (No.)	166
a, b, c, α, β, γ	γ 8.63390(5) Å, 8.63390(5) Å, 21.1398(2) Å, 90°, 90°, 120°
V (Å^3)	1364.41(10)
Z	1
d-space range (Å)	0.58354 - 9.45973
χ^2	2.62 (overall), 2.13 (I11 MAC), 3.47 (HRPD bank1), 22.59 HRPD (bank 2)
R_p	9.58 (overall), 9.58 (I11 MAC), 3.96 (HRPD bank 1), 3.77 (HRPD bank 2)
R_{wp}	7.96 (overall), 12.37 (I11 MAC), 4.66 (HRPD bank1), 4.71 (HRPD bank 2)

$R_p = \frac{\sum|Y_{o,m}-Y_{c,m}|}{\sum Y_{o,m}}$, $R_{wp} = \sqrt{\frac{\sum w_m[Y_{o,m}-Y_{c,m}]^2}{\sum w_m Y_{o,m}^2}}$, $\chi^2 = \frac{\sum w_m[Y_{o,m}-Y_{c,m}]^2}{M-N}$, where $Y_{o,m}$ and $Y_{c,m}$ are the observed and calculated data respectively at data point m; M the number of data points, N the number of parameters, w_m the weighting given to data point m; for counting statistics $w_m = \frac{1}{\sigma(Y_{o,m})^2}$, where $(Y_{o,m})$ is the error in $Y_{o,m}$

References

1. Sansom, H. C.; Whitehead, G. F. S.; Dyer, M. S.; Zanella, M.; Manning, T. D.; Pitcher, M. J.; Whittles, T. J.; Dhanak, V. R.; Alaria, J.; Claridge, J. B.; Rosseinsky, M. J., $AgBiI_4$ as a Lead-Free Solar Absorber with Potential Application in Photovoltaics. *Chem. Mater.* **2017,** *29* (4), 1538-1549.
2. Degen, T.; Sadki, M.; Bron, E.; König, U.; Nénert, G., The HighScore Suite. *Powder Diffr.* **2014,** *29* (S2), S13-S18.
3. Momma, K.; Izumi, F., VESTA: a Three-Dimensional Visualization System for Electronic and Structural Analysis. *J. Appl. Crystallogr.* **2008,** *41* (3), 653-658.
4. Sheldrick, G. M., A Short History of SHELX. *Acta Crystallogr. A* **2008,** *64*, 112-122.
5. Dolomanov, O. V.; Bourhis, L. J.; Gildea, R. J.; Howard, J. A. K.; Puschmann, H., OLEX2: a Complete Structure Solution, Refinement and Analysis Program. *J. Appl. Crystallogr.* **2009,** *42* (2), 339-341.
6. Whittles, T. J.; Burton, L. A.; Skelton, J. M.; Walsh, A.; Veal, T. D.; Dhanak, V. R., Band Alignments, Valence Bands, and Core Levels in the Tin Sulfides SnS, SnS_2, and Sn_2S_3: Experiment and Theory. *Chem. Mater.* **2016,** *28* (11), 3718-3726.
7. Johnston, D. C., Stretched Exponential Relaxation Arising from a Continuous Sum of Exponential Decays. *Phys. Rev. B* **2006,** *74* (18), 184430.
8. Seifert, T.; Jaiswal, S.; Martens, U.; Hannegan, J.; Braun, L.; Maldonado, P.; Freimuth, F.; Kronenberg, A.; Henrizi, J.; Radu, I.; Beaurepaire, E.; Mokrousov, Y.; Oppeneer, P. M.; Jourdan, M.; Jakob, G.; Turchinovich, D.; Hayden, L. M.; Wolf, M.; Münzenberg, M.; Kläui, M.; Kampfrath, T., Efficient Metallic Spintronic Emitters of Ultrabroadband Terahertz Radiation. *Nature Photon* **2016,** *10* (7), 483-488.
9. Joyce, H. J.; Boland, J. L.; Davies, C. L.; Baig, S. A.; Johnston, M. B., A Review of the Electrical Properties of Semiconductor Nanowires: Insights Gained from Terahertz Conductivity Spectroscopy. *Semicond. Sci. Technol.* **2016,** *31* (10), 103003.
10. Chessin, H.; Passarelli, R. E. J.; Vonnegut, B., Investigation of the Pseudobinary Solid Solutions of AgI-CuI by Means of X-Ray Diffraction. *Inorg. Chem.* **1975,** *14* (10), 2551-2552.
11. Mashadieva, L. F.; Aliev, Z. S.; Shevelkov, A. V.; Babanly, M. B., Experimental Investigation of the Ag–Bi–I Ternary System and Thermodynamic Properties of the Ternary Phases. *J. Alloys Compd.* **2013,** *551*, 512-520.
12. Fourcroy, P. H.; Palazzi, M.; Rivet, J.; Flahaut, J.; Céolin, R., Etude du Systeme $AgIBiI_3$. *Mater. Res. Bull.* **1979,** *14* (3), 325-328.
13. Dzeranova, K. B.; Kaloev, N. I.; Bukhalova, G. A., The BiI_3 - AgI System. *Russ. J. Inorg. Chem.* **1985,** *30*, 1700 - 1701.
14. Sansom, H. C.; Longo, G.; Wright, A. D.; Buizza, L. R. V.; Mahesh, S.; Wenger, B.; Zanella, M.; Abdi-Jalebi, M.; Pitcher, M. J.; Dyer, M. S.; Manning, T. D.; Friend, R. H.; Herz, L. M.; Snaith, H. J.; Claridge, J. B.; Rosseinsky, M. J., Highly Absorbing Lead-Free Semiconductor Cu_2AgBiI_6 for Photovoltaic Applications from the Quaternary CuI–AgI–BiI_3 Phase Space. *JACS* **2021,** *143* (10), 3983-3992.
15. Fourcroy, P. H.; Carre, D.; Thevet, F.; Rivet, J., Structure du Tetraiodure de Cuivre(I) et de Bismuth(III), $CuBiI_4$. *Acta Crystallogr. C Struct.* **1991,** *47* (10), 2023-2025.
16. Wyckoff, R. W. G.; Posnjak, E., The Crystal Structures of the Cuprous Halides. *JACS* **1922,** *44* (1), 30-36.
17. Aminoff, G., VII. Über die Kristallstruktur von AgJ. *Z. Kristallogr. Cryst. Mater.* **1922,** *57* (1-6), 180-185.
18. Trotter, J.; Zobel, T., The Crystal Structure of SbI_3 and BiI_3. *Z. Kristallogr. Cryst. Mater.* **1966,** *123* (1-6), 67-72.
19. Bragg, W. H., The Structure of Magnetite and the Spinels. *Nature* **1915,** *95* (2386), 561-561.
20. Bijvoet, J.; Claassen, A.; Karssen, A. In *The Scattering Power of Lithium and Oxygen, Determined from the Diffraction-Intensities of Powdered Lithiumoxide*, Proceedings of the Koninklijke Nederlandse Academie van Wetenschappen, 1926.

21. McQueen, T. M.; Stephens, P. W.; Huang, Q.; Klimczuk, T.; Ronning, F.; Cava, R. J., Successive Orbital Ordering Transitions in $NaVO_2$. *Phys. Rev. Lett.* **2008,** *101* (16), 166402.
22. Pauling, L.; Hoard, J. L., The Crystal Structure of Cadmium Chloride. *Z. Kristallogr. Cryst. Mater.* **1930,** *74* (1-6), 546-551.
23. de Picciotto, L. A.; Thackeray, M. M.; David, W. I. F.; Bruce, P. G.; Goodenough, J. B., Structural Characterization of Delithiated $LiVO_2$. *Mater. Res. Bull.* **1984,** *19* (11), 1497-1506.
